# Water‐Induced Shape‐Locking Magnetic Robots

**DOI:** 10.1002/advs.202405021

**Published:** 2024-07-29

**Authors:** He Lou, Yibin Wang, Yifeng Sheng, He Zhu, Shiping Zhu, Jiangfan Yu, Qi Zhang

**Affiliations:** ^1^ School of Science and Engineering The Chinese University of Hong Kong Shenzhen 518172 China; ^2^ Shenzhen Institute of Artificial Intelligence and Robotics for Society Shenzhen 518172 China

**Keywords:** magnetic actuation, modulus switchable polymer, small‐scale soft robot, smart materials

## Abstract

Untethered magnetic soft robots capable of performing adaptive locomotion and shape reconfiguration open up possibilities for various applications owing to their flexibility. However, magnetic soft robots are typically composed of soft materials with fixed modulus, making them unable to exert or withstand substantial forces, which limits the exploration of their new functionalities. Here, water‐induced, shape‐locking magnetic robots with magnetically controlled shape change and water‐induced shape‐locking are introduced. The water‐induced phase separation enables these robots to undergo a modulus transition from 1.78 MPa in the dry state to 410 MPa after hydration. Moreover, the body material's inherent self‐healing property enables the direct assembly of morphing structures and magnetic soft robots with complicated structures and magnetization profiles. These robots can be delivered through magnetic actuation and perform programmed tasks including supporting, blocking, and grasping by on‐demand deformation and subsequent water‐induced stiffening. Moreover, a water‐stiffening magnetic stent is developed, and its precise delivery and water‐induced shape‐locking are demonstrated in a vascular phantom. The combination of untethered delivery, on‐demand shape change, and water‐induced stiffening properties makes the proposed magnetic robots promising for biomedical applications.

## Graphical abstract

This work presents the design, fabrication, and application of magnetic soft robots capable of shape locking through water‐induced phase separation. The robots’ modulus transitions from 1.78 to 410 MPa upon hydration. The flexibility in the dry state and rigidity in the wet state enable the delivery, reconfiguration, and deployment of these robots for various applications.

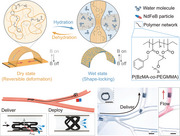

## 1 Introduction

Magnetic soft robots exhibit untethered, rapid, and reversible actuation and locomotion in response to magnetic fields.^[^ ^1^, ^2^, ^3^, ^4^ ^]^ These robots with programmed magnetization profiles can navigate through biofluid (e.g., blood, gastric juices, mucus) and confined spaces (e.g., duct and vessel),^[^ ^5^, ^6^, ^7^, ^8^, ^9^, ^10^, ^11^, ^12^, ^13^, ^14^ ^]^ making them promising candidates for biomedical applications, such as drug delivery and minimally invasive surgery.^[^ ^15^, ^16^, ^17^, ^18^, ^19^, ^20^, ^21^ ^]^ Typically, these robots are constructed using body materials with a low Young's modulus, such as hydrogels and poly(dimethylsiloxane) (PDMS).^[^ ^22^ ^]^ Benefiting from their softness, magnetic soft robots exhibit active or passive deformability and mechanical compliance matching with soft tissues, which ensures their fundamental function (actuation, locomotion) and safe interaction with soft tissue. However, the softness also constrains the robots' ability to apply or withstand substantial forces. In nature, some soft biological structures like tongues, trunks, and tentacles can adjust their stiffness to mimic rigid skeletal structures to perform various tasks not achievable by common soft biological structures.^[^ ^23^, ^24^, ^25^ ^]^ Inspired by these biological structures, we anticipate that endowing magnetic soft robots with switchable stiffness could give rise to new functions and promote their active interaction with environments.^[^ ^26^, ^27^, ^28^ ^]^

Modulus switchable materials such as shape memory polymer (SMP),^[^ ^29^, ^30^ ^]^ low melting point alloys (LMPAs),^[^ ^31^, ^32^ ^]^ electroactive gel,^[^ ^33^ ^]^ and magnetoactive fluid,^[^ ^34^, ^35^ ^]^ which can transition modulus in response to external stimuli such as heat, electric field, or magnetic field, have been used for constructing soft robots or actuators with switchable modulus (Table S1, Supporting Information).^[^ ^36^ ^]^ For example, a magnetic actuator exhibiting a significant modulus change from 2.4 MPa to 2.9 GPa as the temperature decreases from 100 to 20 °C was developed using ferric‐oxide‐infused thermoresponsive SMPs.^[^ ^29^ ^]^ Magnetic robotic catheters based on LMPA are developed for transluminal procedures.^[^ ^31^ ^]^ However, the application of these materials in biomedical scenarios still faces challenges. The use of thermoresponsive SMP or LMPA requires heating which may risk harming the tissue. Besides, the plasticizing effect of water and the disruption of hydrogen bonding interactions among polymer chains have a weakening effect on most polymers, including SMPs, limiting the application of conventional thermoresponsive SMP in scenarios like stents that demand sustained high‐modulus states for stable fixation.^[^ ^37^, ^38^, ^39^ ^]^ The use of LMPA requires encapsulation to avoid the exposure of toxic LMPA to the physiological environment. To overcome these limitations, there is a need for a material system that stiffens in response to ambient stimuli and maintains mechanical stability in physiological environments.

Water emerges as a promising gentle stimulus due to its biocompatibility, and ubiquitous presence in the physiological environment.^[^ ^40^, ^41^, ^42^ ^]^ Water‐induced stiffening SMPs, used as the body materials for magnetic robots, can avoid potential safety concerns and are expected to achieve an increase in modulus in aqueous environments. Herein, we propose water‐induced, shape‐locking magnetic (W‐SLM) robots composed of modulus‐switchable polymer poly(benzyl methacrylate‐*co*‐poly(ethylene glycol) methyl ether methacrylate) (P(BzMA‐*co*‐PEGMMA)) and embedded neodymium–iron–boron (NdFeB) magnetic particles. In the dry state, the robot exhibits flexible elastomeric properties with an initial modulus of 1.78 MPa, allowing reversible deformation and movement under magnetic field. Upon water absorption, the robot changes into a rigid shape‐locking state with a modulus of 410 MPa, due to phase separation induced by water absorption. Besides the significant modulus change, the NdFeB/P(BzMA‐*co*‐PEGMMA) composite material also exhibits self‐healing properties in the dry state, enabling direct assembly of complex structures from building blocks. The self‐healing capability and modulus change grant this material advanced processability and functionality. We demonstrate the programming of morphing structures with complicated magnetization profiles, the design and fabrication of W‐SLM robots with functionalities such as supporting, grasping, and blocking, and also explore their potential as polymer stents. We anticipate that the combination of magnetic actuation and shape locking could enable applications across various fields.

## 2 Results

### 2.1 Design of W‐SLM Robot

**Figure 1:**
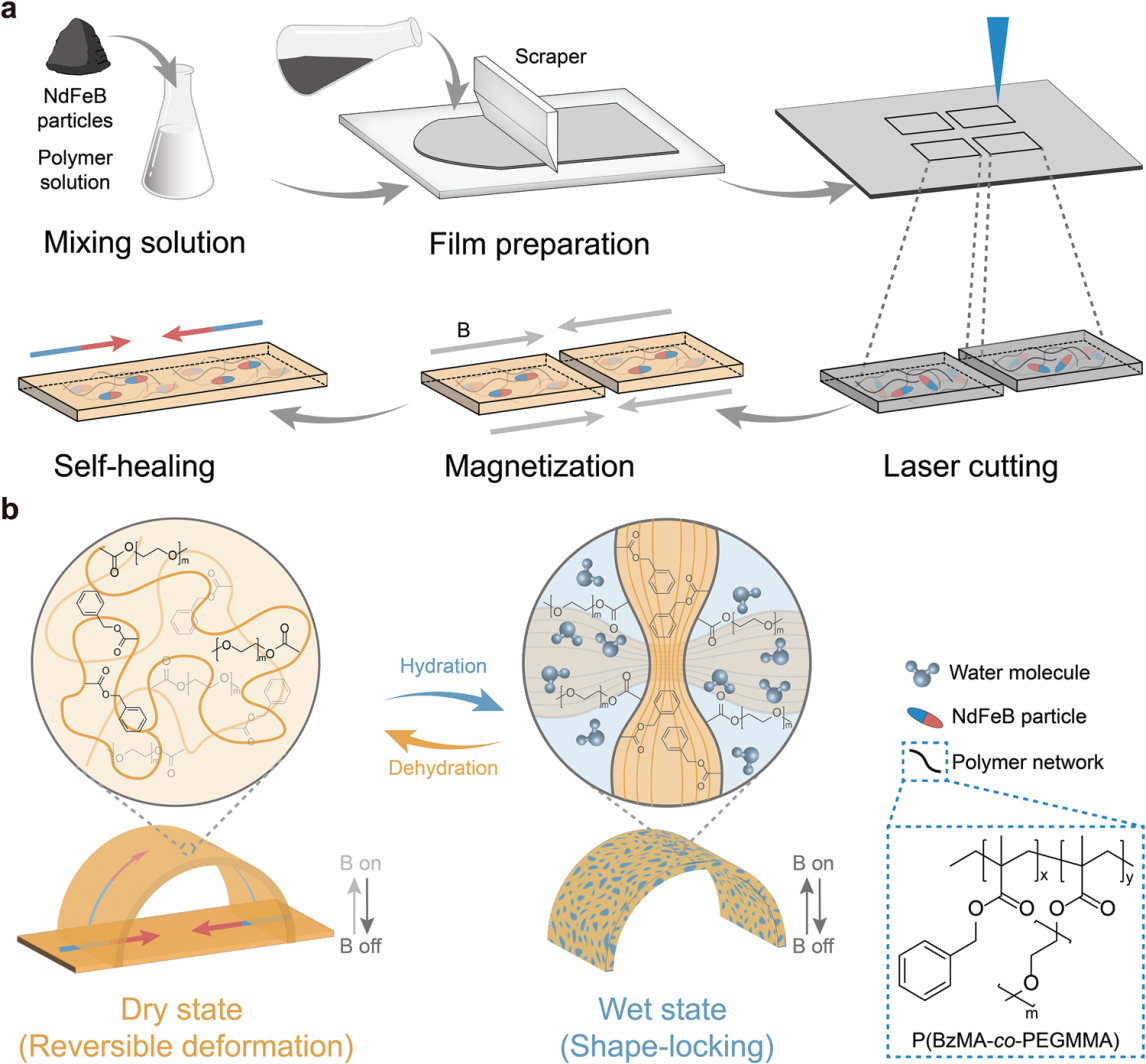
Scheme of water‐induced, shape‐locking magnetic robots. a) Schematic diagram showing the fabrication process of NdFeB/P(BzMA‐*co*‐PEGMMA) composite film and W‐SLM robot. The polymer solution exhibits moderate viscosity, and can be spread onto a PET release film. After evaporating the acetone, the composite film was obtained. The robot was constructed using two identical rectangular composite films obtained through CO_2_ laser cutting, each magnetized to saturation in opposite directions and subsequently assembled through a self‐healing process. b) The working mechanism of the W‐SLM robot. The P(BzMA‐*co*‐PEGMMA) in the composite film is homogenous at dry state. The PEG side chains act as plasticizers and are evenly dispersed between the main chains. The hydration of hydrophilic PEG side chains induces phase separation, in which PEG and water form one phase, while the hydrophobic main chain is expelled to form another phase, resulting in a transition from rubbery to glassy state.

Ideally, the W‐SLM robots, constructed using water‐driven, modulus switchable polymer materials as body materials, are supposed to achieve versatile, rapid deformations and movements in a dry or early wet state, which require that the body materials have specific mechanical properties (Young's modulus ranging between 0.1 and 10 MPa and an elongation at break exceeding 200%).^[^ ^43^, ^44^, ^45^, ^46^ ^]^ Besides, these robots should be capable of exhibiting a large increase in modulus and locking into specific shapes in a fully wet state to execute the corresponding functions. Therefore, we established a strategy for designing W‐SLM robots, including fabrication and working mechanism (**Figure** **1**). Drawing upon prior research,^[^ ^47^ ^]^ we have selected a comb‐like copolymer, P(BzMA‐*co*‐PEGMMA), as the body material for our robots, which can be easily synthesized through free‐radical solution polymerization (Figure S1, Supporting Information). The copolymer's main chains predominantly comprise hydrophobic P(benzyl methacrylate) (PBzMA), offering good chain mobility and a moderate glass transition temperature (*T*_g_). Its dangling side chains consist of hydrophilic polyethylene glycol (PEG), a widely used polymer in the biomedical field, renowned for its antifouling properties, biocompatibility, and flexibility. The NdFeB/P(BzMA‐*co*‐PEGMMA) composite film (≈350 um) was fabricated by incorporating NdFeB particles (about 5 µm) into a viscous P(BzMA‐*co*‐PEGMMA) acetone solution, followed by spreading the mixture solution and subsequent acetone evaporation. Scanning electron microscopy (SEM) analysis and the corresponding element mapping of the composite film confirm the uniform distribution of NdFeB particles within the polymer matrix (Figure S2, Supporting Information). Various W‐SLM robots can be designed and fabricated through a straightforward process involving laser cutting, magnetization with the desired magnetic profiles, and self‐healing of building blocks of the NdFeB/P(BzMA‐*co*‐PEGMMA) composite film (Figure 1a).

As shown in Figure 1b, the working mechanism of a W‐SLM robot with a simple structure (magnetic profile shown in Figure 1a) is exemplified. In its dry state, the presence of dangling PEG side chains which serve as plasticizers, would reduce the copolymer's *T*_g_, thus contributing to the low modulus of the robot. The favorable Young's modulus of robots facilitates actuation and meets the mechanical compliance requirement to passively adapt to the shape of environment during delivery. Upon applying a magnetic field, the robot undergoes programmed shape change. During the hydration process, water molecules would penetrate the hydrophobic segments and stabilize within PEG‐rich domains, triggering phase separation and volume expansion within the polymer matrix, thereby leading to increased volume and rigidity of the robot.^[^ ^37^, ^48^, ^49^ ^]^ Notably, the favorable chain mobility of hydrophobic segments and the dangling PEG chains significantly increase the degree of phase separation and further inhibit unlimited swelling of the PEG chain.^[^ ^50^ ^]^ Consequently, a substantial increase in modulus compared to the dry state can be achieved. Upon removal of the magnetic field, the deformation shape can be securely locked with high stiffness during the later stage of the hydration process, allowing the robot to actively exert force on the environment or specific objects, enabling expanded functions.

### 2.2 Characterization of Water‐Stiffening Magnetic Polymer Composite

**Figure 2:**
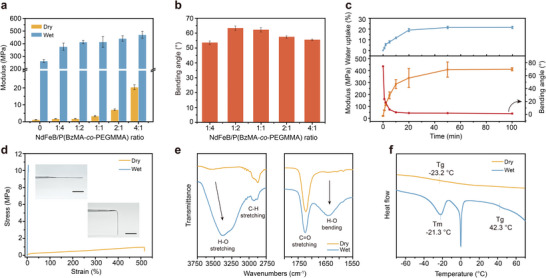
Characterization of water‐stiffening magnetic polymer composite. a) Young's modulus of NdFeB/P(BzMA‐*co*‐PEGMMA) composite films with different NdFeB particles to P(BzMA‐*co*‐PEGMMA) weight ratio (0, 1:4, 1:2, 1:1, 2:1, 4:1) at dry and wet states (after immersing in water for 100 min). b) Actuation performance (evaluated by the bending angle) of dehydrated composite films with different NdFeB to P(BzMA‐*co*‐PEGMMA) weight ratios. The composite films have identical size and thickness. c) Water uptake, tensile modulus, and bending angle of the composite film (weight ratio of 2:1) as a function of water immersion time (blue line: water uptake; orange line: modulus; red line: bending angle). The bending of the composite film is driven by a 20 mT magnetic field. d) Tensile test results of composite film (weight ratio of 2:1) at dry and wet states. The inset figures show that the composite film is soft at dry state, and becomes rigid at wet state. Scale bars: 2 cm. e) FTIR spectra and f) DSC curves of composite film (weight ratio of 2:1) at dry and wet states.

The incorporation of NdFeB into the polymer matrix imparts magnetic responsiveness, while simultaneously stiffening the polymer matrix as fillers. As the ratio between NdFeB to P(BzMA‐*co*‐PEGMMA) varied from 0 to 4:1, the Young's modulus of the composite films at dry state increased from 1.20 to 20.3 MPa (**Figure** **2**a and Table S2 (Supporting Information)), with stretchability decreasing from 700% to 45% (Figure S3a, Supporting Information). The increase in stiffness is attributed to the inherent rigidity of the inorganic NdFeB particles.^[^ ^51^ ^]^ Compared to the neat polymer, the composite films show a reduction in stretchability, which can be ascribed to the relatively larger particle size (5 µm) and the poor bonding between particles and the polymer matrix, hindering the effective transfer of applied stress from the NdFeB particles to the polymer matrix.^[^ ^51^, ^52^ ^]^ As the NdFeB concentration increases, the particles tend to aggregate within the polymer matrix, creating cavities between particles that affect particle–matrix adhesion and further reduce stretchability (Figure S3b, Supporting Information). The increase in stiffness could pose a challenge to efficient magnetic actuation. A similar trend has also been observed in hydrated composite films (Figure 2a and Figure S3c (Supporting Information)). To determine the appropriate composition of the polymer composite ensuring magnetic actuation, a bending test of the composite films under a magnetic field of 20 mT was conducted (Figure S3c, Supporting Information). The composite films with a NdFeB‐to‐P(BzMA‐*co*‐PEGMMA) weight ratio of 1:2 exhibited the best magnetic response as a result of the interplay between the mechanical properties and magnetization (Figure 2b).

To better understand the water‐induced stiffening process, a quantitative investigation was conducted on the tensile and actuation performance of the composite film over time after water immersion (Figure 2c and Figure S4a,b (Supporting Information)). In the dry state, the composite film exhibited a soft and flexible elastomeric behavior with a Young's modulus of 1.78 ± 0.21 MPa and an elongation at a break of 592 ± 107% (Figure 2d). Throughout the hydration process, the composite film experienced a rapid increase in water uptake and modulus, while the increase rate showed a gradual decrease due to impedance in water diffusion. Equilibrium was reached after 100 min, with a water uptake of 21 wt%. A linear correlation between water absorption and modulus was observed throughout the process (Figure S4c, Supporting Information). Concurrently, the bending angle and stretchability of the composite film rapidly decreased in the early stage, reaching 2.0° after 20 min and remaining essentially unchanged thereafter. Upon complete hydration, the elastomer transformed into a highly rigid, shape‐locking state, with the modulus increasing to 410 ± 13 MPa (modulus switch ratio exceeding 200).

To provide a more comprehensive understanding of the mechanism behind the water‐induced stiffening process, a series of tests were conducted. Figure 2e shows Fourier transform infrared spectroscopy (FTIR) spectra of the composite film before and after hydration. The presence of water‐characteristic peaks (H─O vibration) in the dry state was attributed to the hygroscopic nature of PEG. Upon exposure to water, the intensity of H─O stretching (3200–3700 cm^−1^) and H─O bending (1639 cm^−1^) bands significantly increased, indicating the introduction of water in composite film (Figure 2f). Moreover, the H─O stretching peak shifted to lower wavenumbers, attributed to the disruption of the water–PEG interaction by the water hydrogen bond network.^[^ ^53^ ^]^ The oxygen atom in water has a lower negative charge density than the ethereal oxygen atom in PEG due to the inductive donating effect of alkyl groups in PEG, resulting in a stronger ability to attract electrons. Consequently, the hydrogen bonds between water molecules are stronger than those between water and PEG molecules, weakening the H─O covalent bond and inducing the redshift of water molecules.^[^ ^54^, ^55^ ^]^ Subsequently, water removed PEG, the plasticizer, from the hydrophobic main chains, leading to phase separation into two distinct phases: the PEG–water phase and the hydrophobic main chain phase, as confirmed by differential scanning calorimeter (DSC) measurements. In the dry state, the composite film shows a low *T*_g_ of −23.3 °C, resulting in low modulus and good bending ability. In the wet state, the melting peak of the PEG–water phase (*T*_m_ = −21.3 °C) emerged,^[^ ^56^ ^]^ along with the glass transition of rearranged hydrophobic main chain phase (*T*_g_ = 42.3 °C), contributing to high modulus and shape‐locking effect for composite film. Small‐angle X‐ray scattering (SAXS) measurements also confirmed the phase separation phenomenon with the appearance of scattering peaks in the lower *q*‐region after hydration, attributed to the difference in electron density between the PEG–water phase and the hydrophobic main chain phase (Figure S5, Supporting Information). Notably, although the concentration of NdFeB particles significantly affected the mechanical properties of the composite film at dry state, it had minimal impact on the degree of phase separation (Figure S6 and Table S2, Supporting Information).

### 2.3 Exploiting Self‐Healing for Programming Magnetization Profile

The NdFeB/P(BzMA‐*co*‐PEGMMA) composite films exhibited remarkable self‐healing capabilities, primarily driven by polymer chain interdiffusion at the interface (Figure S7a, Supporting Information).^[^ ^57^, ^58^, ^59^, ^60^, ^61^ ^]^ The reptation model indicates that the repair time depends on the molecular weight dependence (*T*_r_ ∝ *M* ^3^), suggesting that the dangling PEG chains with low molecular weight are more flexible and beneficial to high mobility chain segment motion, thus facilitating self‐healing.^[^ ^62^, ^63^, ^64^, ^65^ ^]^ Additionally, the dangling PEG chains act as a plasticizer, reducing the *T*_g_ (excess free volume), and therefore enhancing diffusion.^[^ ^59^, ^66^, ^67^ ^]^ To demonstrate the self‐healing capability of the polymer composite, a composite film was cut into two parts, magnetized with different magnetization directions, and completely healed at 70 °C (Figure S7b, Supporting Information). No remaining cracks were observed at the fracture interface of the healed composite film, even after hydration (Figure S7c,d, Supporting Information). Furthermore, the magnetization profile was retained after healing (Figure S7e, Supporting Information). This self‐healing property enables the fabrication of W‐SLM robots through the assembly and healing of magnetized building blocks, thereby achieving the simultaneous construction of complex structures and programming of magnetization profiles. In contrast to conventional soft robots predominantly reliant on covalently cross‐linked elastomers such as PDMS, whose fabrication is confined to injection molding, our approach facilitates the production of small‐scale soft robots with intricate 3D structures.^[^ ^68^ ^]^

**Figure 3:**
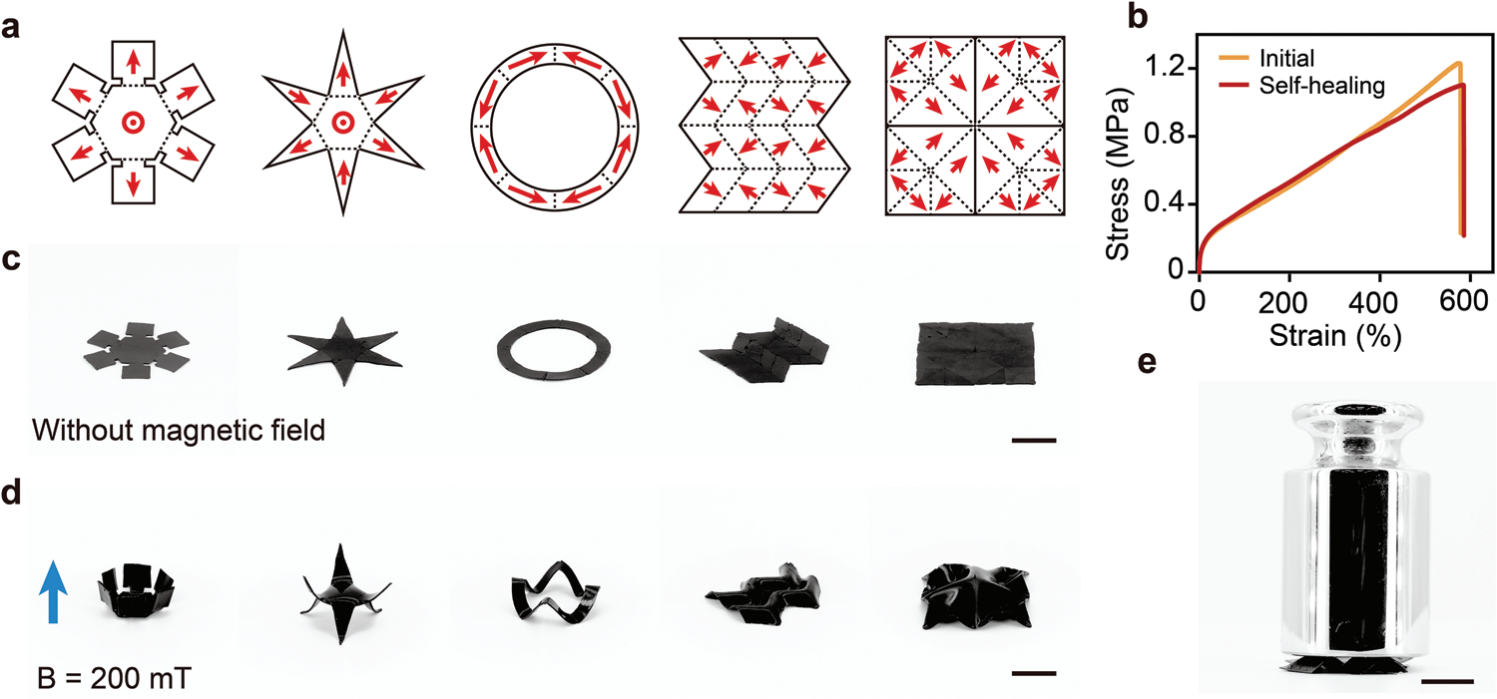
Morphing structures fabricated through self‐healing of magnetized building blocks. a) Magnetization profiles of magnetic morphing structures assembled from NdFeB/P(BzMA‐*co*‐PEGMMA) composite building blocks (dashed lines: the joint parts; red arrows: the magnetization directions of each building block). b) Tensile test results of the composite film before and after self‐healing. c) Digital photos of 2D magnetic morphing structures in original configurations without external magnetic field. d) Digital photos of magnetic morphing structures in deformed configurations under magnetic actuation. e) The deformed magnetic morphing structure with a Miura‐Ori pattern supports a weight of 200 g after shape locking (after immersing in water for 100 min). Scale bars: 1 cm.

Employing the self‐healing‐enabled fabrication process, a series of magnetic morphing structures with 2D geometries and designed magnetization profiles were successfully produced (**Figure** **3**a). Remarkably, these self‐healed magnetic morphing structures retained their original modulus and stretchability post a 12 h self‐healing process at 70 °C in a dry state, with self‐healing efficiencies reaching 96.9% (Figure 3b and Table S3 (Supporting Information)). After hydration, the healed composite film maintained a similar modulus to the original sample but exhibited reduced stretchability (Figure S8a and Table S3, Supporting Information). Furthermore, the composite film also exhibited excellent cyclic performance and recyclability, which are highly beneficial for practical manufacturing and applications (Figure S8b,c, Supporting Information).

The deformation of these magnetic morphing structures under magnetic actuation was explored. To mitigate the influence of magnetic forces, a permanent cylindrical magnet with a diameter of 50 mm was employed. Initially configured in 2D (Figure 3c), these structures swiftly reconfigured into 3D shapes upon exposure to a magnetic field of ≈200 mT, demonstrating rapid magnetic response (Figure 3d). Subsequent removal of the magnetic field prompted the morphing structures to revert to their flat shape, due to the elastic properties inherent in the polymer composite. Notably, the shape‐locked morphing structure with a Miura‐Ori pattern was capable of supporting a weight of 200 g after fully hydration, despite the robot itself weighing only 0.21 mg, demonstrating its magnetic‐induced shape change and the water‐induced shape locking (Figure 3e). Additionally, the shape‐locked morphing structure can be unlocked by simply removing the water through oven drying or by raising the temperature to 70 °C (Figures S9 and S10, Supporting Information).

### 2.4 Designing Shape‐Locking Magnetic Robots

Through the integration of magnetic delivery, on‐demand shape reconfiguration, and shape‐locking, we anticipate that these robots could execute intricate tasks such as supporting, grasping, and blocking. Robotic supports, grippers, and helices have been designed and fabricated to demonstrate their delivery and deployment within the simulated water‐filled vascular environment. To preserve the deformability of the robot during transit and deployment, we regulated the time of water‐induced stiffening by applying a sacrificial PEG layer on the robot's surface (Figure S11, Supporting Information).

**Figure 4:**
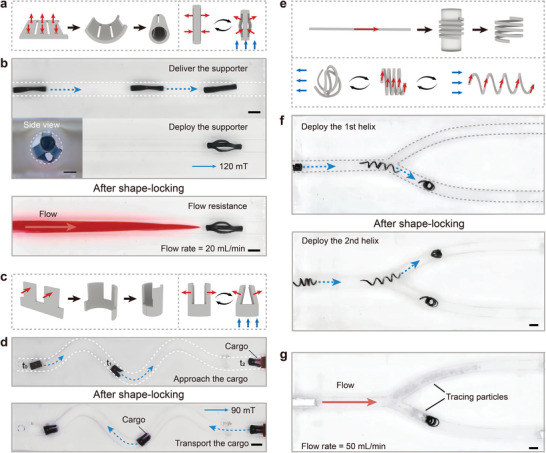
Delivery and deployment of shape‐locking magnetic robots. a) Fabrication and magnetic actuation of the magnetic robotic support (red arrows: magnetization direction; blue arrows: direction of external magnetic field). b) Delivery and deployment of a magnetic robotic support in a straight channel (inset: side view of the deployed robotic stent inside the channel; white dashed lines: the outline of the tube; blue dashed arrow: the moving direction of the robot; orange arrow: the flow direction). Scale bar: 2 mm. c) Fabrication and magnetic actuation of the magnetic robotic gripper. d) Cargo transportation in a curved channel achieved by a magnetic robotic gripper. e) Fabrication and magnetic actuation of the magnetic robotic helix. f) Independent delivery and deployment of magnetic robotic helices in a Y‐shaped channel. g) Flow blocking achieved by the magnetic‐field‐induced coiling of a magnetic robotic helix. Scale bars: 5 mm.

The cylindrical robotic support was constructed from a magnetized composite film that had been precisely patterned through laser cutting. The film was then rolled around a cylindrical template and healed to form a slitted cylinder support (**Figure** **4**a). When subjected to external magnetic fields along its longitudinal axis, the robotic support can contract into a thin cylindrical shape or expand to a barrel shape, depending on the direction of the magnetic field. During delivery, the robotic support can be smoothly guided through the channel in a contracted state. Upon reaching the target position, it can be expanded on‐demand to support the channel wall by applying a magnetic field with a strength of around 120 mT in its longitudinal direction. Subsequently, the deployed robotic support underwent modulus increase and volume expansion through water‐induced stiffening, gradually fixing its shape and position. After shape‐locking, the magnetic field was removed, and a water flow with a rate of 20 mL min^−1^ was introduced into the channel. The deployed robotic support demonstrated resistance to the flow and maintained its initial position, indicating the stability of the deployed robotic support (Figure 4b and Video S1 (Supporting Information)).

In order to facilitate grasping and cargo transportation tasks, we have fabricated a robotic gripper with a two‐finger design by rolling and healing a laser‐cut composite film. The film was magnetized to encode a specific magnetization profile that would enable magnetic‐field‐controlled opening and closing (Figure 4c). During a cargo retrieval task, the robotic gripper was initially navigated through a tortuous channel in an open state. Upon reaching the target object, the gripper closes its fingers to securely grasp the cargo under a magnetic field with a strength of around 90 mT. Subsequent shape‐locking ensured that the cargo was firmly held within the closed gripper, enabling its robust transportation (Figure 4d and Video S2 (Supporting Information)). While the opening and closing actions of the gripper were synchronized with the magnetic delivery of the robotic gripper, maintaining its closed state during locomotion presents a notable challenge. However, the water‐induced shape locking allows for the decoupling of the grasping function from the locomotion of the robotic gripper, which significantly enhances the efficiency of cargo transportation.

The shape‐locking feature can also be used in designing robots with blocking function, particularly in tubular environments. To this end, we have developed robotic helices tailored for such applications. These helices were fabricated by wrapping composite film wires around glass tubes, followed by stress relaxation at a temperature of 70 °C. Actuated by external permanent magnets, these helices can either elongate or self‐coil into dense aggregates, depending on the direction of the external magnetic field (Figure 4e). The elongation capability of the robotic helix facilitated its efficient delivery within a confined channel. Upon reaching the targeted area, reversing the magnetic field induced the helix to entangle into a stable aggregate. Based on this magnetic actuation mechanism, we have demonstrated the sequentially obstruction of a Y‐shaped channel utilizing two robotic helices (Figure 4f and Video S3 (Supporting Information)). Notably, the manipulation of the second helix did not interfere with the stability of the first, as it forms a rigid aggregate after stiffening. Our investigations have confirmed that, with water‐induced shape‐locking, the independent control of multiple robotic helices for multiple‐target implementations can be effectively realized. Furthermore, the blocking performance of the robotic helix was validated in the channel. Following the deployment of a robotic helix on one side of the channel and subsequent shape locking, a water flow of 50 mL min^−1^ was introduced, along with tracing particles injected into the flow. As shown in Figure 4g and Video S3 (Supporting Information), the deployed robotic helix successfully blocked all tracing particles. The controlled locomotion, on‐demand deformation, and shape‐locking capabilities of the robotic helices make them promising candidates for replacing conventional spring coils for endovascular embolization, thereby offering a minimally invasive and biocompatible approach.

### 2.5 Delivery and Deployment of Water‐Stiffening Magnetic Stent In Vitro

**Figure 5:**
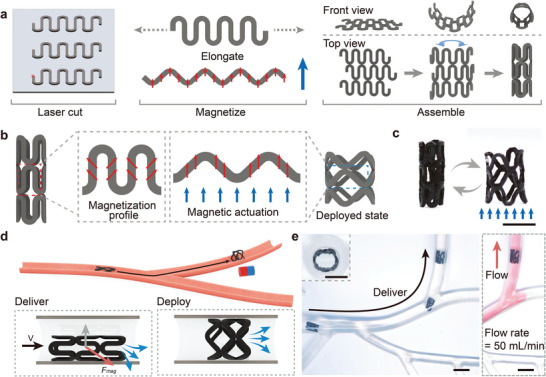
The design, fabrication, and deployment of a water‐stiffening magnetic stent. a) Fabrication process of the water‐stiffening magnetic stent (red arrows: the magnetization direction; blue arrows: the direction of the external magnetic field). b) The magnetic actuation mechanism of the water‐stiffening magnetic stent. c) Digital photos of the magnetic stent in the initial and deformed states. Scale bar: 5 mm. d) Schematics showing the working mechanism of the water‐stiffening stent. The stent can be delivered in vascular systems actuated by an external permanent magnet and can expand at the targeted region. e) Delivery and deployment of the water‐stiffening magnetic stent. After shape locking, the stent can resist a flow of 50 mL min^−1^. Scale bar: 10 mm (inset: the cross‐sectional view of the deployed stent; scale bar: 5 mm).

Endowed with features of active magnetic delivery, programmed reconfiguration, and shape‐locking capabilities, W‐SLM robots demonstrate significant potential for biomedical applications. In pursuit of such applications, we have designed a water‐stiffening magnetic stent. As illustrated in **Figure** **5**a, the water‐stiffening magnetic stent was assembled from wavy building blocks. Initially, these blocks were elongated into an expanded state and magnetized. Following magnetization, they reverted to their original shape and were subsequently arranged into a 2D mesh and then wrapped around a cylindrical support for healing. Afterward, the stent was carefully removed from the support. Upon exposure to an external magnetic field aligned with the longitudinal direction of the stent, magnetic torques acted on the beams of the wavy blocks, driving the expansion of the stent, as shown in Figure 5b. In a dry state, this magnetic stent was able to undergo reversible shape transitions between its original and expanded states when subjected to the magnetic field (Figure 5c).

The delivery of the stent through blood vessels can be accomplished through the magnetic forces generated by a magnetic field gradient. Upon reaching the target region, the stent can be expanded by applying a magnetic field along its longitudinal direction (Figure 5d). The deployment of the magnetic stent was verified in a blood vessel phantom. The stent was introduced into the phantom in a contracted state and then delivered with an external permanent magnet. Throughout the delivery process, the stent experienced slight expansion due to the presence of the magnetic field. Upon reaching the target site, a magnetic field with a local strength of 120 mT was applied to achieve full expansion of the stent. After expansion and shape‐locking, the stent securely supported the vessel wall. Notably, the deployed stent showed the capability to withstand a water flow rate of 50 mL min^−1^ (Figure 5e and Video S4 (Supporting Information)). As a proof of concept, we have demonstrated that such water‐stiffening magnetic stents have the potential to operate as untethered medical robots.

To improve the biocompatibility of the polymer composite, NdFeB particles with silica coating are also prepared (Figure S12, Supporting Information). Cell viability tests were conducted on P(BzMA‐*co*‐PEGMMA), NdFeB/P(BzMA‐*co*‐PEGMMA), and silica‐coated NdFeB/P(BzMA‐*co*‐PEGMMA). The results show that human umbilical vein endothelial cells (HUVECs) exhibit over 95% viability after coculture with P(BzMA‐*co*‐PEGMMA) and silica‐coated NdFeB/P(BzMA‐*co*‐PEGMMA) for 72 h, showing no observable decrease compared to the control group (Figure S13a,b, Supporting Information), which indicate that silica coating can improve the biocompatibility of the polymer composite. According to the hemolytic assay, the polymer and polymer composite exhibit hemocompatibility comparable to the negative control, showing no observable hemolytic effect (Figure S13c, Supporting Information).

## 3 Conclusion

We have successfully developed a water‐stiffening magnetic polymer composite, enabling the creation of a series of modulus‐switchable magnetic soft robots. These robots showcase untethered navigation, rapid and reversible shape reconfiguration, and a unique water‐induced shape‐locking feature. Notably, the shape‐locking ability significantly enhances the functions of magnetic soft robots. Leveraging these advancements, we have engineered magnetic soft robots capable of being delivered and deployed in a compliant state and subsequently locking their configurations for specific tasks such as supporting, grasping, and blocking. Additionally, the self‐healing property of the polymer composite is instrumental in constructing magnetic soft robots with intricate 3D structures from magnetized components, while also allowing for the simultaneous programming of their magnetization profiles. We have demonstrated the design, fabrication, and deployment of water‐stiffening magnetic stents in vascular phantom, verified the potential for applying the proposed composite materials to construct small‐scale soft robots for biomedical applications.

## 4 Experimental Section

### Preparation of P(BzMA‐*co*‐PEGMMA)

P(BzMA‐*co*‐PEGMMA) was synthesized via thermally induced free radical polymerization. In a 250 mL round‐bottom flask, 40 g of benzyl methacrylate (98%, Energy Chemical), 20 g of poly(ethylene glycol) methyl ether methacrylate (*M*_w_ ≈950, Aladin), 360 mg of 2,2′‐azobis(2‐methylpropionitrile), and 60 mL of dimethylformamide were sequentially added. The mixture was stirred at 65 °C for 24 h under a nitrogen atmosphere. After cooling to room temperature, the P(BzMA‐*co*‐PEGMMA) was precipitated by adding water. This reprecipitation process was repeated 3 times for purification, followed by drying at 60 °C.

### Preparation of the NdFeB/P(BzMA‐*co*‐PEGMMA) Composite Film

Typically, 10 g of P(BzMA‐*co*‐PEGMMA) was dissolved in 15 mL of acetone (0.67 g mL^−1^), and 5 g of NdFeB magnetic particles (5 µm, MQFP‐15‐7, Magnequench) was added. The mixture was thoroughly vortexed to ensure an even distribution of NdFeB particles. The resultant NdFeB/P(BzMA‐*co*‐PEGMMA) solution was then spread onto a polyethylene terephthalate (PET) release film and left to air‐dry overnight.

### Preparation of the Silica‐Coated NdFeB/P(BzMA‐*co*‐PEGMMA) Composite Film

The silica‐coated NeFeB magnetic particles were prepared following a reported method.^[^ ^3^ ^]^ The following process was same as the preparation of NdFeB/P(BzMA‐*co*‐PEGMMA) composite film.

### Preparation of W‐MSL Robots

W‐MSL robot building units were laser‐cut using a CO_2_ laser platform (Fusion Edge, EpilogLaser). These units were then magnetized using a magnetizer (MA 2030, Shenzhen Jiuju Industrial Equipment Co. Ltd.). The W‐MSL robot was assembled and healed at 70 °C for 12 h. The PEG sacrificial layer was coated by immersing the W‐SLM robot into melted PEG1000, followed by the removal of excess PEG, and left to cool at room temperature.

### Water Absorption Rate

The NdFeB/P(BzMA‐*co*‐PEGMMA) composite film at dry state was cut into rectangular pieces (20 mm × 5 mm) and their dry weight (*m*_dry_) was measured, followed by immersing into deionized (DI) water at room temperature for a different time. The water on the surface was wiped out with wet tissue. The wet weight was measured (*m*_wet_). The water absorption rate was calculated by the following Equation (1)

(1) $$\mathrm{Water}\mathrm{uptake}\left(\%\right)=\left(m_{\mathrm{wet}}-m_{\mathrm{dry}}\right)/m_{\mathrm{dry}}\times 100\%$$

### Mechanical Properties

Tensile tests were performed on Instron testing machine equipped with a 100 N load cell at room temperature (Instron, 5967). The sample was cut into a rectangular shape (20 mm × 5 mm, thickness of 350 µm). All the samples at wet state were tested after immersing in water for 100 min.

### Preparation of Phantoms

The injection molding technique was employed to fabricate the PDMS phantoms with the desired lumen geometries, following a reported method.^[^ ^69^ ^]^ The positive molds with the required lumen shapes were first 3D printed and secured within a rectangular polymethyl methacrylate (PMMA) mold. PDMS (mass ratio 10:1, Sylgard 184, Dow Inc.) was poured into the PMMA mold and cured at 70 °C. The PDMS was then carefully cut open, and the 3D‐printed positive molds were removed to create negative molds. These molds were filled with melted paraffin wax, cooled to solidify, and fixed in rectangular PMMA molds, followed by pouring PDMS (mass ratio 10:1) and curing at room temperature for 48 h. The PDMS phantoms with hollow lumen channels were heated to melt and remove the wax. Finally, the hollow PDMS phantoms were ultrasonically treated in ethanol to remove any wax residuals.

### Cell Viability Test

To evaluate the biocompatibility of the polymer and polymer composite, cell viability tests were conducted using HUVECs. Cells were seeded in a 48‐well plate at a density of 40 000 cells per well and cultured in an incubator at 37% with 5% CO_2_. Rectangular films of P(BzMA‐*co*‐PEGMMA), NdFeB/ P(BzMA‐*co*‐PEGMMA), and silica‐coated NdFeB/P(BzMA‐*co*‐PEGMMA) (5 mm × 5 mm × 0.4 mm) were added to the wells 24 h after cell seeding. For the control group, no additional materials were added. After coculture for 24, 48, and 72 h, cell viability was determined with the Calcein/PI cell viability/cytotoxicity assay kit (Beyotime). Stained cells were imaged with a fluorescence microscope (Nikon, ECLIPSE Ti2) and counted with ImageJ (version 2.1.0).

### Hemolytic Assay

Hemolytic assays were conducted using fresh anticoagulated porcine blood (Guangzhou Hongyang Co. Ltd.). The blood sample (2 mL) was first diluted with 10 mL phosphate‐buffered saline (PBS). The diluted blood was then centrifuged at 1500 rpm for 10 min to isolate red blood cells (RBCs). After washing 3 times, the isolated RBCs were resuspended in 2 mL of PBS. For the negative control, 200 µL of diluted blood was added to 400 µL of PBS in a 48‐well plate. For the positive control, 200 µL of diluted blood was added to 400 µL of DI water. Rectangular films of P(BzMA‐*co*‐PEGMMA), NdFeB/P(BzMA‐*co*‐PEGMMA), and silica‐coated NdFeB/P(BzMA‐*co*‐PEGMMA) (5 mm × 5 mm × 0.4 mm) were added to the mixture of 200 µL of diluted blood and 400 µL of PBS. The samples were then incubated at 37 °C for 4 h. After incubation, the mixtures were centrifuged (1500 rpm, 5 min). The supernatants were diluted 30 times with PBS and their absorbance was measured with UV–vis spectrophotometer (Shimadzu UV‐1900). The hemolysis rate was calculated with the following Equation (2)

(2) $$\mathrm{Hemolysis}\;\left(\%\right)=\left(\frac{\mathrm{Ab}s_{\mathrm{sample}}\;-\;\mathrm{Ab}s_{\mathrm{negative}\;\mathrm{control}}}{\mathrm{Ab}s_{\mathrm{positive}\;\mathrm{control}}\;-\;\mathrm{Ab}s_{\mathrm{negative}\;\mathrm{control}}}\right)\;\times \;100\%$$

### Characterization

^1^H‐Nuclear magnetic resonance spectroscopy (^1^H‐NMR) spectra were conducted on a Bruker Avance Neo 500. Chemical shifts were calibrated by the residual solvent peak as reference (CDCl_3_ = 7.2 ppm). Optical microscope images were taken by Nikon ECLIPSE Ti2, magneto‐optical microscope image was taken by polar magneto‐optical Kerr microscope (Evico Magnetics GmbH). SEM with energy‐dispersive spectroscopy (EDS) analyses were carried out with a CIQTEK SEM 5000 microscope with an Oxford X‐Max^N^ 50 EDS detector. Attenuated total reflectance‐FTIR spectra were obtained using Thermo Scientific Nicolet iS10 in the region of 400–4000 cm^−1^ at room temperature. DSC measurement experiments were conducted on a calorimeter (TA Instruments DSC 2500) at a programmed ramp of 10 °C min^−1^ from −80 to 80 °C under a nitrogen atmosphere. SAXS was carried on a Cu Kα X‐ray radiation source (Xeuss 2.0, Xenocs). Dynamic mechanical test was conducted with a dynamic mechanical analyzer (Q850, TA Instruments). The temperature ranged from 30 to 85 °C and the amplitude was fixed at 0.1% strain, 1 Hz.

## Conflict of Interest

The authors declare no conflict of interest.

## Author Contributions

H.L. and Y.W. contributed equally to this work. H.L., Y.W., J.Y., and Q.Z. conceived the idea and designed the research. H.L., Y.W. performed the experiments, analyzed data, and drafted the paper, Y.S., H.Z., S.Z., J.Y., and Q.Z. commented and revised the paper. J.Y. and Q.Z. supervised the project, and all authors reviewed the paper.

## Supporting information

Supporting Information

Supplemental Video 1

Supplemental Video 2

Supplemental Video 3

Supplemental Video 4

## Data Availability

The data that support the findings of this study are available from the corresponding author upon reasonable request.
