# Supplementary material for: Water‐Induced Shape‐Locking Magnetic Robots
Source: Adv Sci (Weinh). 2024 Jul 29;11(36):2405021. doi: 10.1002/advs.202405021 (PMC11423202; doi:10.1002/advs.202405021)
Supplement: Supplementary file 1 — Supporting Information [file ADVS-11-2405021-s001.docx]

Supporting Information

Water-induced Shape-Locking Magnetic Robots

He Lou, Yibin Wang, Yifeng Sheng, He Zhu, Shiping Zhu, Jiangfan Yu* and Qi Zhang*

These authors contributed equally to this work: He Lou, Yibin Wang.

**Supplementary Figures and Figure Captions**
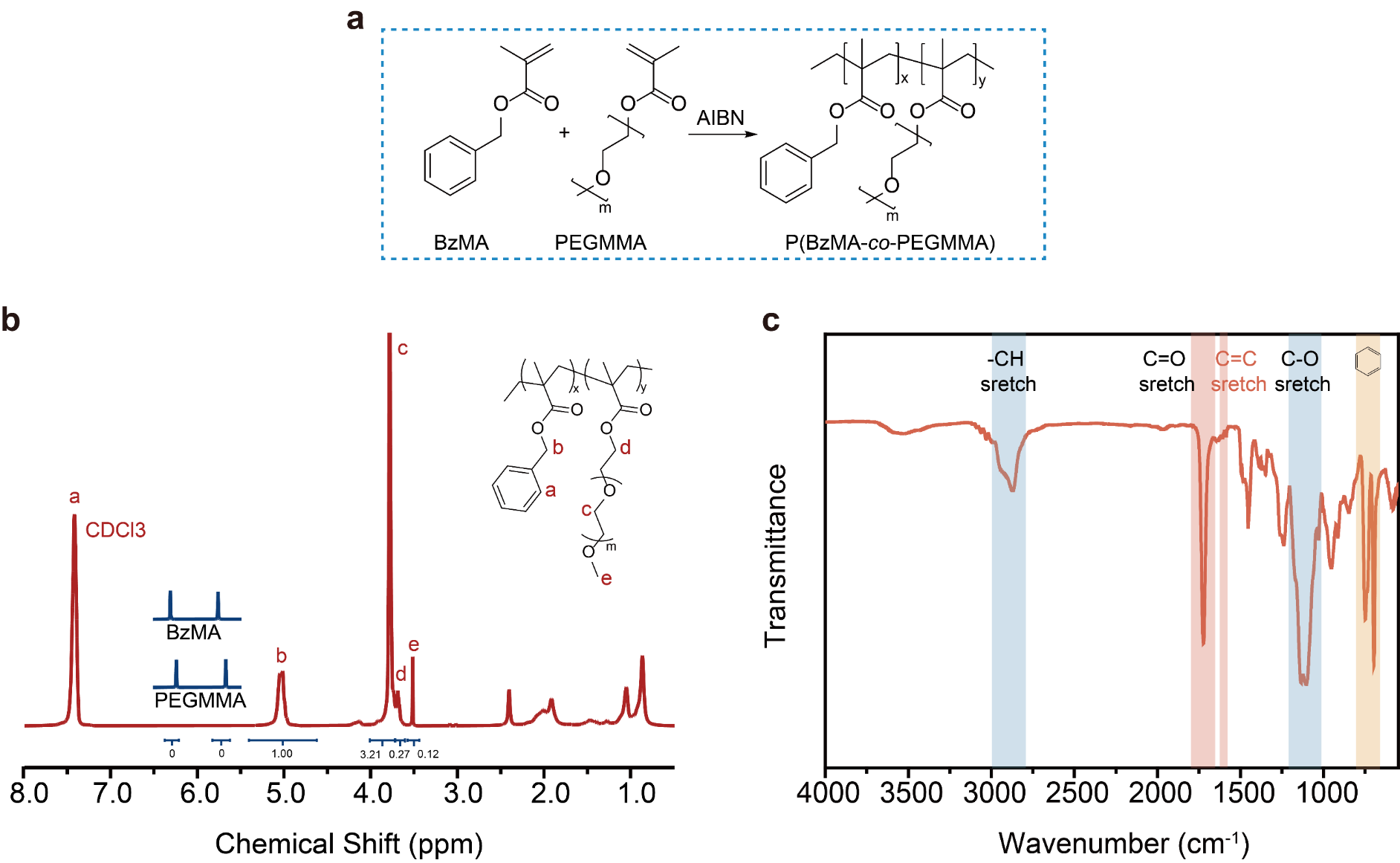


**Figure S1.** a) Synthesis of P(BzMA-*co*-PEGMMA). BzMA and PEGMMA served as monomers in a 2:1 weight ratio. b) ^1^H NMR spectra of copolymer (Inserted blue line: ^1^H-NMR spectra of monomers). c) FTIR spectra of copolymer. Blue block: characteristic peaks mainly present in PEGMMA; Yellow block: characteristic peak of BzMA; Red block: characteristic peaks contained in both BzMA and PEGMMA.

The ^1^H NMR spectra of P(BzMA-*co*-PEGMMA) are shown in Figure S1b. The precise ratio of x:y can be calculated by their corresponding area of the peaks, $Sb/2: Se/3=25:2$, while the m can be calculated by $Sc/4: Se/3\approx20$. The FTIR spectra indicate the characteristic peaks of P(BzMA-*co*-PEGMMA), consisting of benzene ring bending at 697 cm^−1^, C$-$H hoop at 748 cm^−1^ (characteristic peaks of BzMA), C$-$H and C$-$H_2_ stretching at near 2800-3100 cm^−1^, C$-$O stretching at 1134 cm^-1^ (characteristic peaks of PEG), and C$=$O stretching at 1723 cm^-1^.^[1,2]^ The characteristic peak of monomer, C$=$C stretching is not observed in the FTIR spectra of P(BzMA-*co*-PEGMMA), suggesting low concentrations or even no remaining of BzMA and PEGMMA in the final products. Both FTIR and ^1^H NMR analysis confirm the successful polymerization with minimal monomer residues.

**
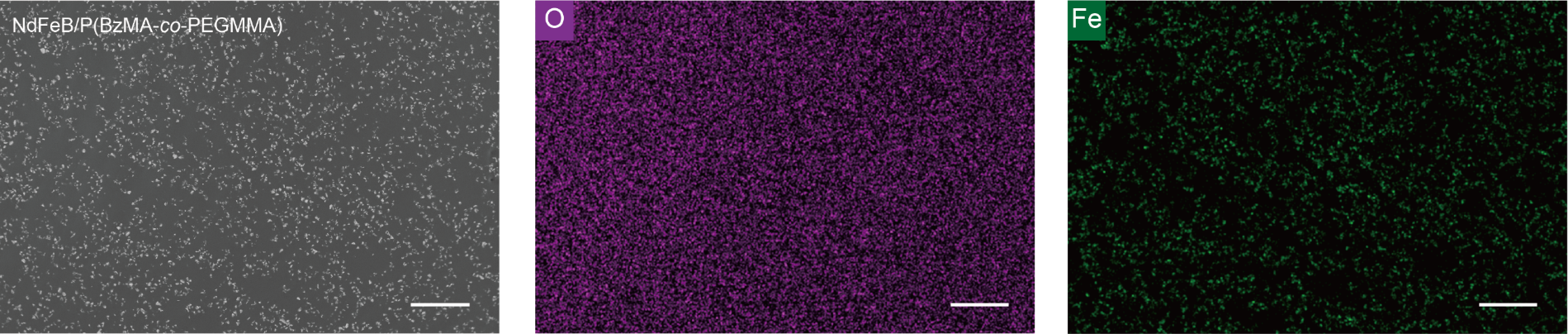
**

**Figure S2.** SEM image and corresponding element mapping of the surface of NdFeB/P(BzMA-*co*-PEGMMA) composite film (weight ratio of 2:1). Scale bar: 100 μm.

**
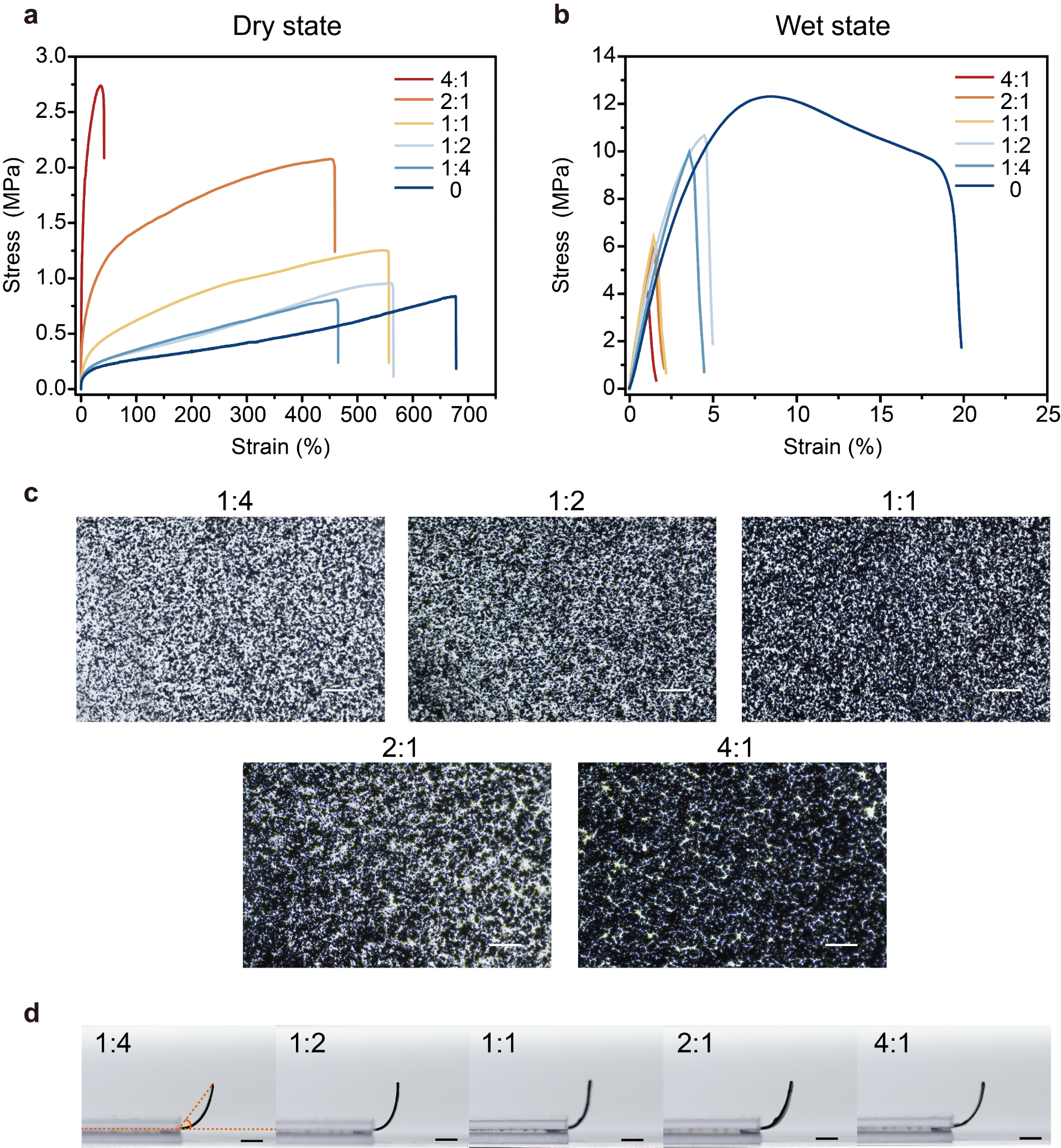
**

**Figure S3.** a) Tensile test results of composite films with different NdFeB particles to P(BzMA-*co*-PEGMMA) weight ratio (0, 1:4, 1:2, 1:1, 2:1, 4:1) at dry and b) wet state (after immersing in water for 100 min). b) Optical microscope of composite films with different NdFeB to P(BzMA-*co*-PEGMMA) weight ratios. Scale Bar: 100 μm. c) Optical images showing the bending of the dehydrated composite films with different NdFeB to P(BzMA-*co*-PEGMMA) weight ratios. Scale Bar: 3 mm.


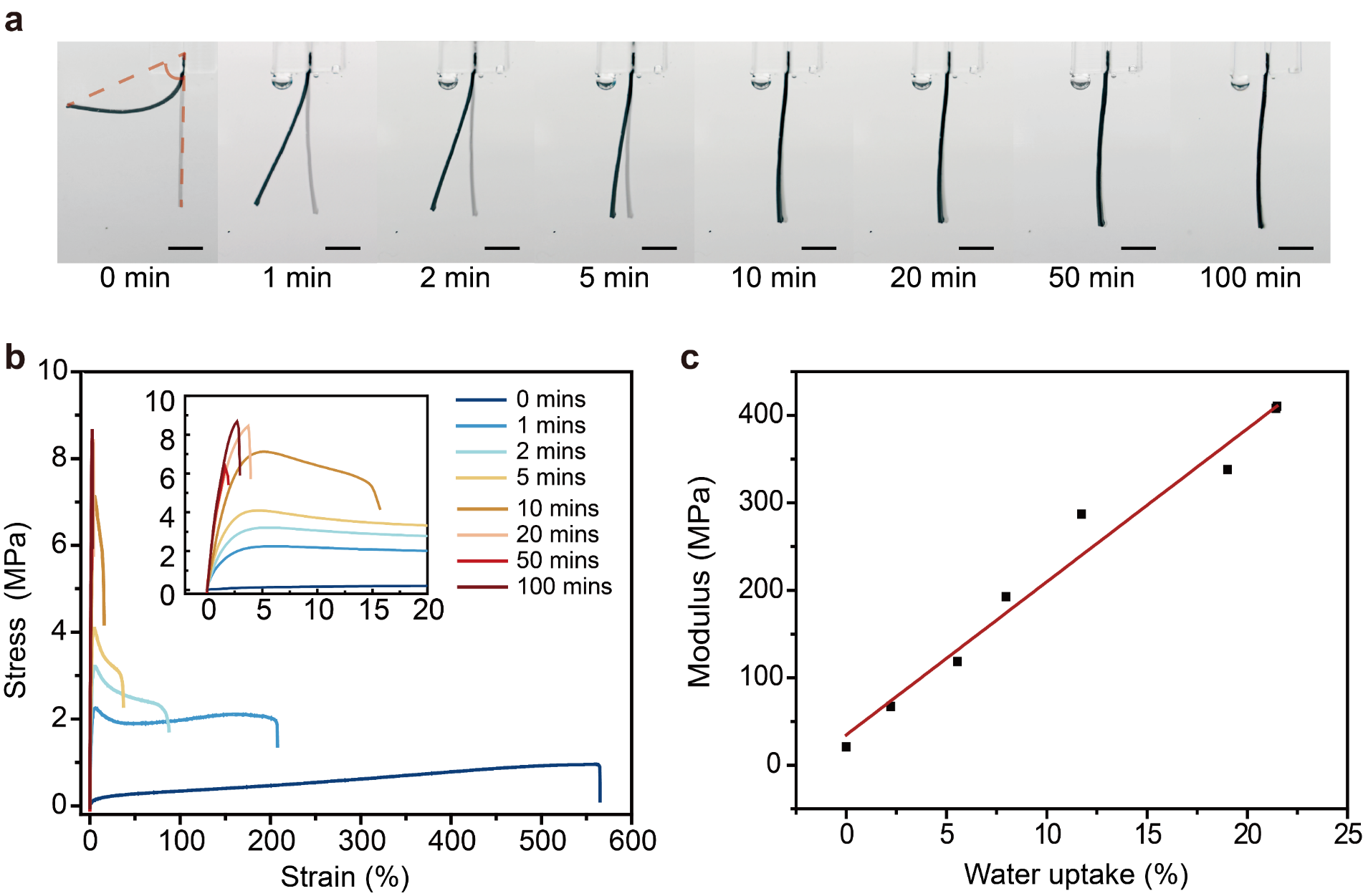


**Figure S4.** a) Optical images showing bending of NdFeB/P(BzMA-*co*-PEGMMA) composite film (weight ratio of 2:1) at different time scales after soaking. Scale bar: 2 mm. b) Tensile test results of composite films at different time scales after soaking. c) The plot of modulus to water uptake shows a linear relationship between modulus and water uptake (R^2^= 0.97).

**
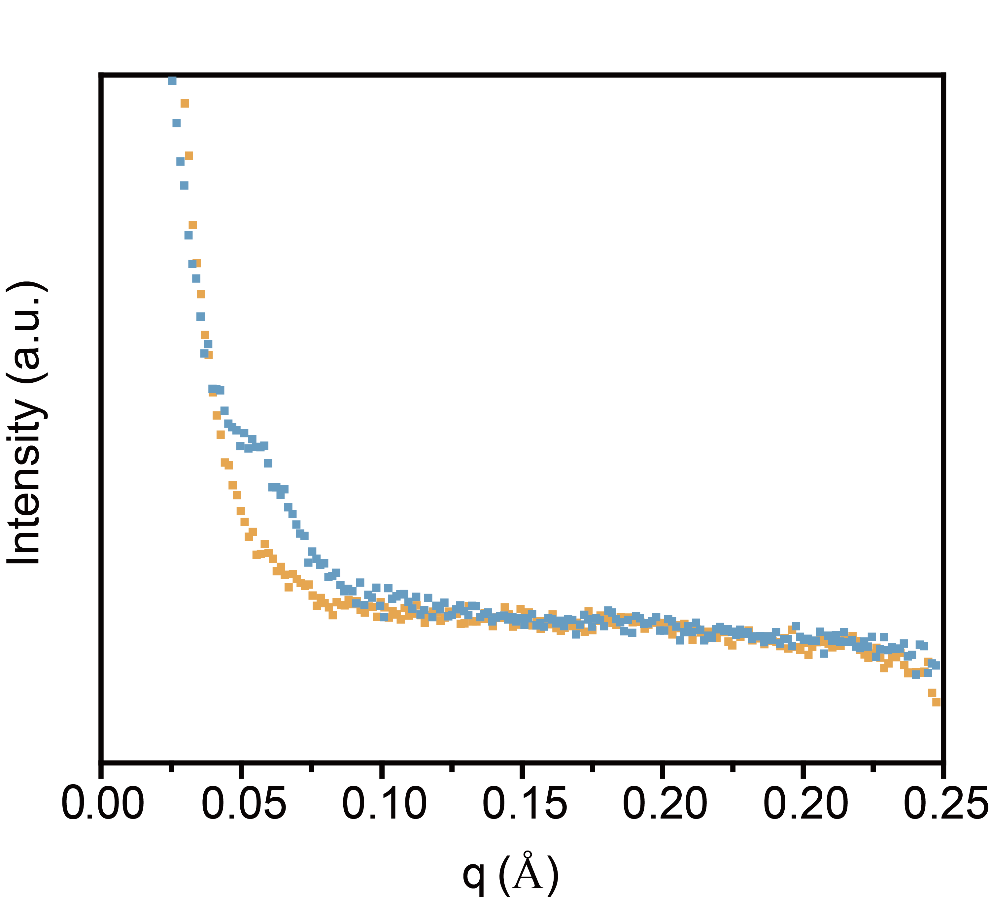
**

**Figure S5.** SAXS analysis of NdFeB/P(BzMA-*co*-PEGMMA) composite films with weight ratio of 2:1 (Orange line: at dry state; blue line: at wet state).

The scattering pattern of the NdFeB/P(BzMA-*co*-PEGMMA) composite film at wet state exhibits interaction peaks at scattering angles maximum of 0.05855$Å^{-1}$, which means the average interparticle distances resulting from short-range order interactions is calculated by $d={2\pi}/q=10.7 nm$. The radius of gyration, Rg, of the scattering particles, can be estimated by the Guinier’s equation $I(q) \propto(exp -q^{2} {Rg}^{2}/3)$, where $I(q)$ is the scattering intensity, q is the scattering vector. The Guinier approximation posits that a linear relationship should be observed at low q region with a slope of $-{Rg}^{2}/3$.^[3]^ The radius of gyration values (Rg) of about 8.3 nm for NdFeB/P(BzMA-*co*-PEGMMA) composite film at wet state.

**
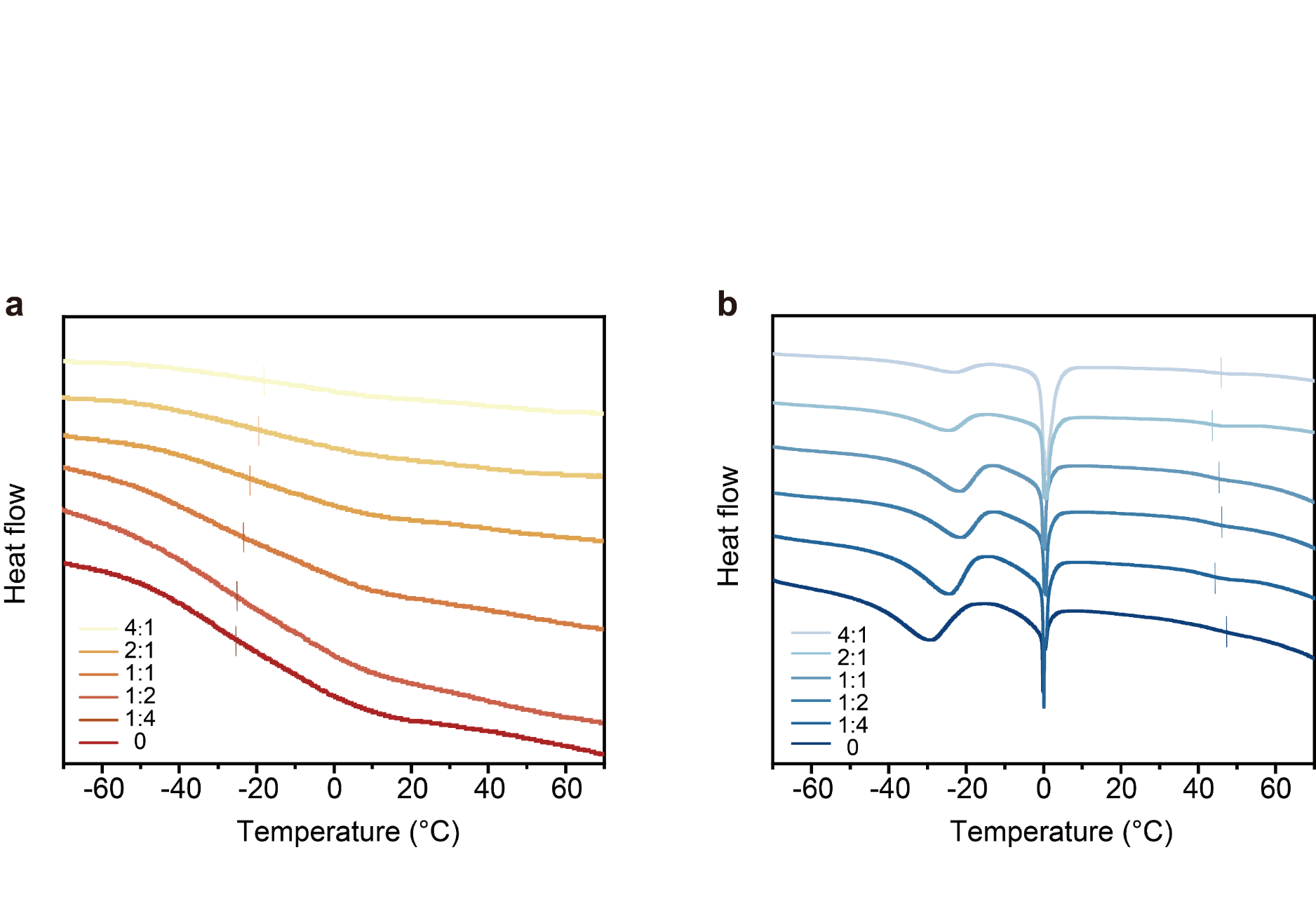
**

**Figure S6.** a) DSC curves of NdFeB/P(BzMA-*co*-PEGMMA) composite films with different NdFeB particles to P(BzMA-*co*-PEGMMA) weight ratio (0, 1:4, 1:2, 1:1, 2:1, 4:1) at dry and b) wet state (after immersing in water for 100 min).

In the dry state, DSC data show that an increase in the concentration of magnetic powder results in a slight increase in the *T_g_* of the composite film. This is attributed to the hindrance of polymer chain motion and diffusion caused by the presence of magnetic particles. However, in the wet state, the *T_g_* of the hydrophobic main chain phase, which plays a more significant role in modulus, does not undergo substantial changes with varying concentrations of magnetic particles. Consequently, for samples with different concentrations of magnetic particles, the *T_g_* variation before and after water absorption is not significant. Therefore, the addition of magnetic particles does not greatly impact the degree of phase separation.

**
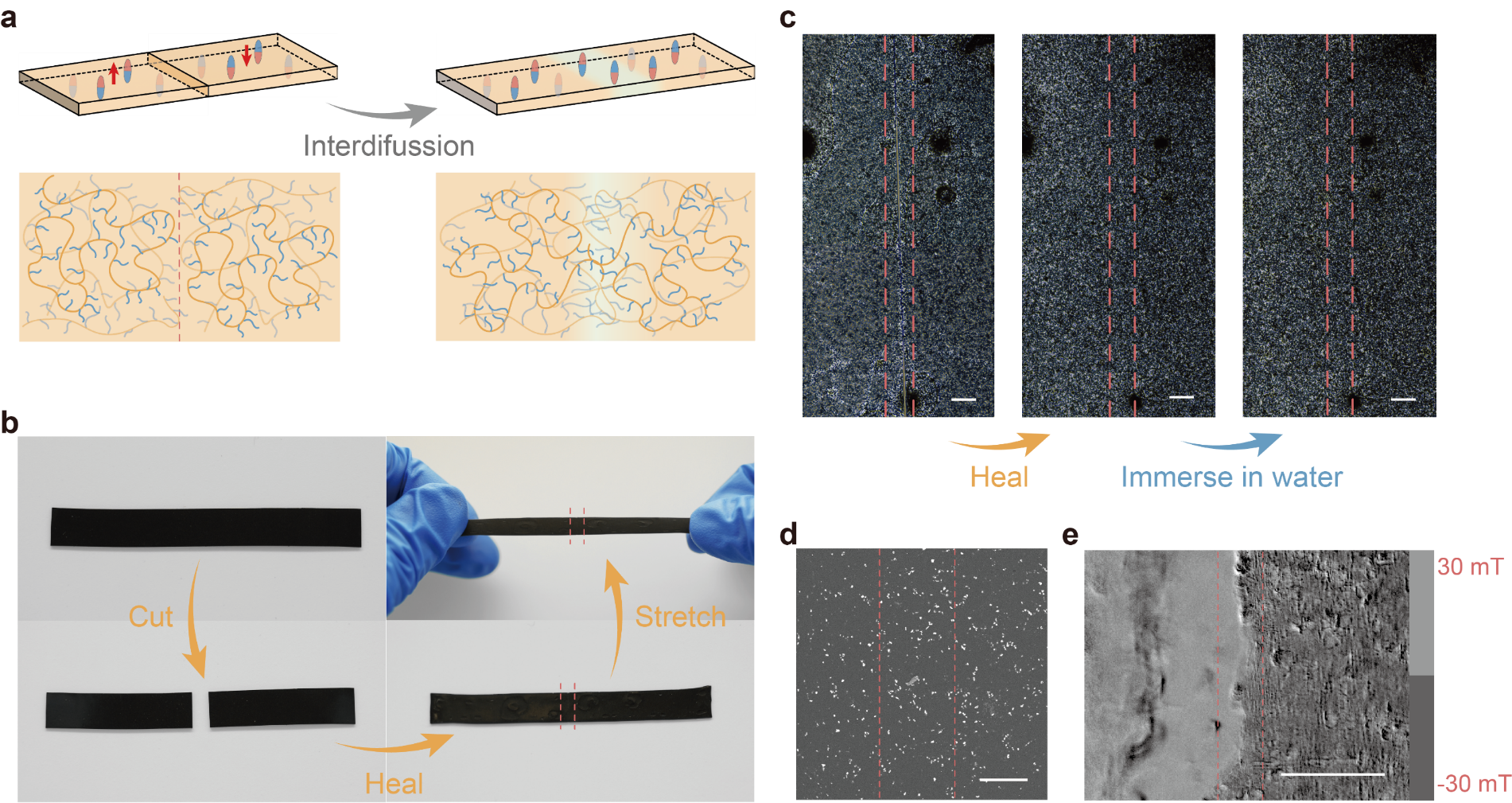
**

**Figure S7.** a) Schematic diagram of possible healing mechanism in NdFeB/P(BzMA-*co*-PEGMMA) composite film with weight ratio of 2:1. b) Digital photos of the self-healing process of the composite film. c) Optical microscope images of the damaged, healed composite films at dry state and healed composite film at wet state. Scale Bar: 200 μm. d) SEM image and e) magneto-optical microscope image of the healed composite film. Scale Bar: 100 μm.

**
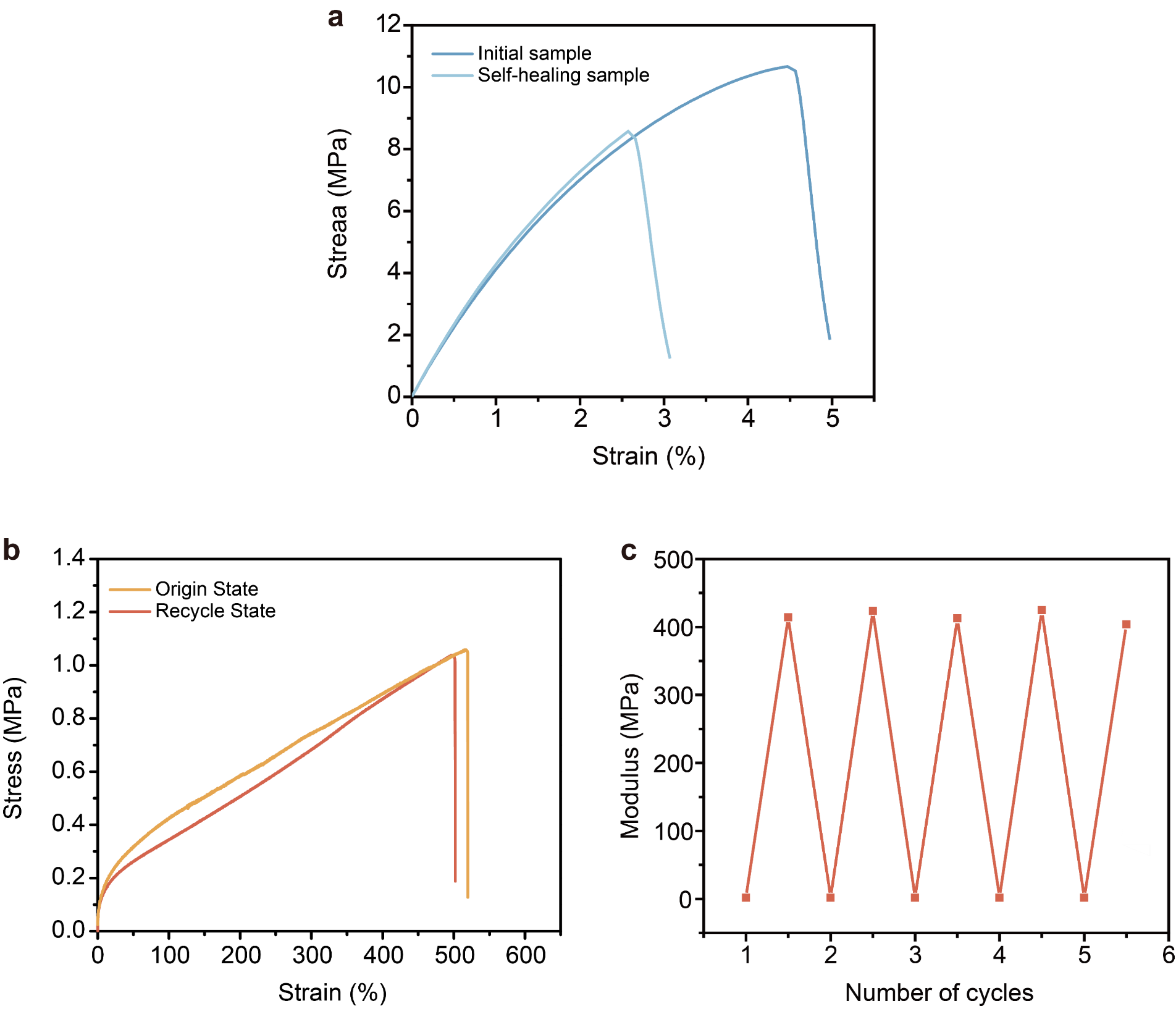
**

**Figure S8.** a) Tensile test results of the original and self-healed NdFeB/P(BzMA-*co*-PEGMMA) composite films (weight ratio of 2:1) after hydration (immersing in water for 100 min). b) Tensile test results of the original and recycled composite films at dry state. c) The reversible mechanical strength of the composite film in successive hydration-dehydration cycles.

In the dry state, the self-healing sample of NdFeB/P(BzMA-*co*-PEGMMA) composite film displays remarkable self-healing efficiency, showcasing mechanical properties almost identical to the initial sample. However, a notable reduction in elongation at break occurs when the healed sample changes to a hydrated state. Here is a possible reason: the entanglement of the dangling PEG short chains dominates the healing process, so when the composite film absorbs water, the phase separation causes the rearrangement of the polymer main chains and side chains, exposing defects. Moreover, the composite film exhibits excellent recyclability, achieved by dissolving the discarded film in acetone, spreading the NdFeB/P(BzMA-*co*-PEGMMA) solution onto a PET release film, and evaporating the solvent. The resulting recycled samples mirror the original's mechanical properties, confirming the film's exceptional recyclability. Minor variations in the tensile test curve may arise from the rearrangement of magnetic particles during recycling. This outstanding self-healing capability and recyclability offer versatile manufacturing options for composite film. Apart from laser cutting, hot pressing, or template casting can be used to create various complex models. Additionally, the film displays remarkable reversibility between soft (dry) and rigid states (wet). After undergoing six hydration-dehydration cycles, the film's modulus remains almost unchanged, indicating its good cyclic performance.


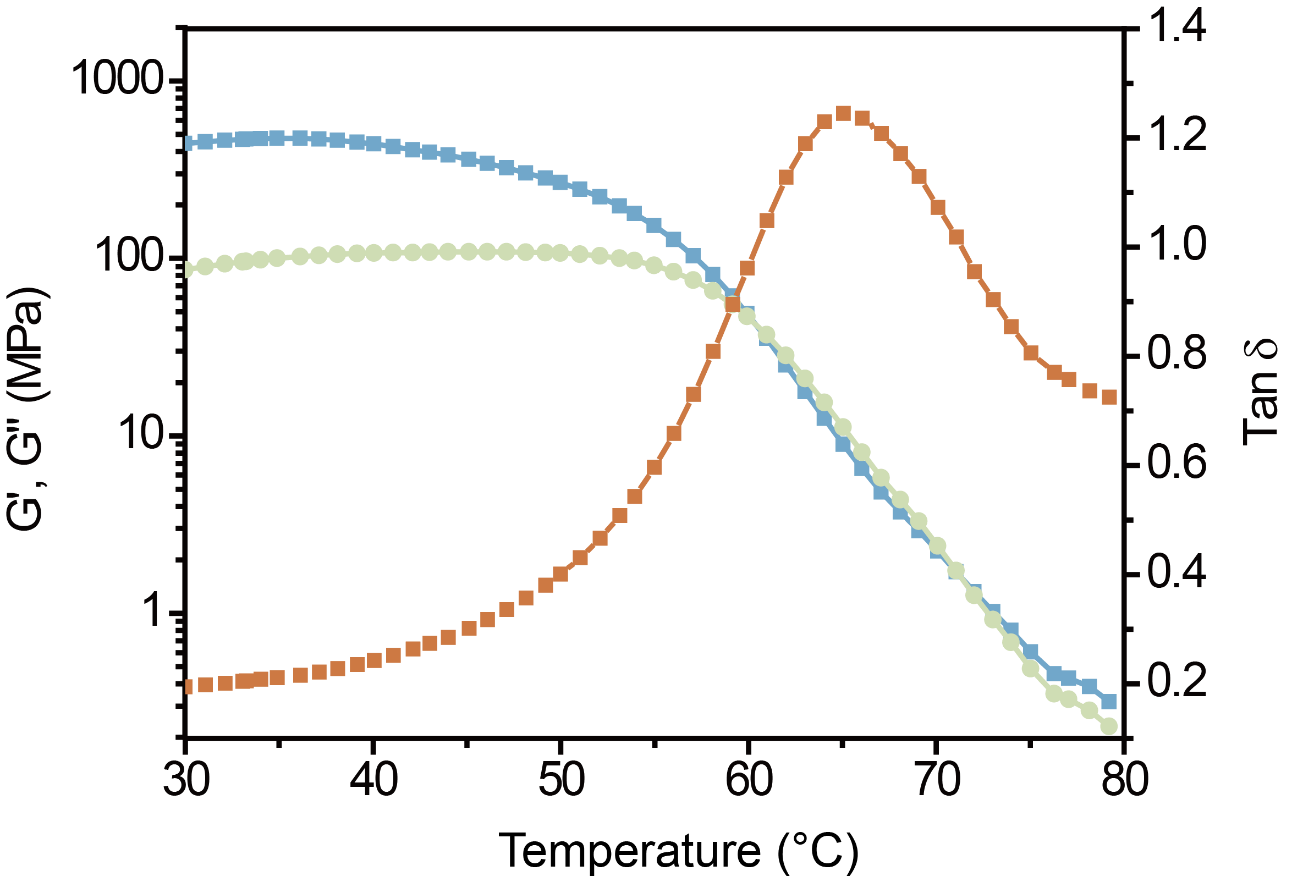


**Figure S9.** Dynamic mechanical storage modulus, loss modulus, and tan δ of the hydrated NdFeB/P(BzMA-*co*-PEGMMA) composite film versus temperature.


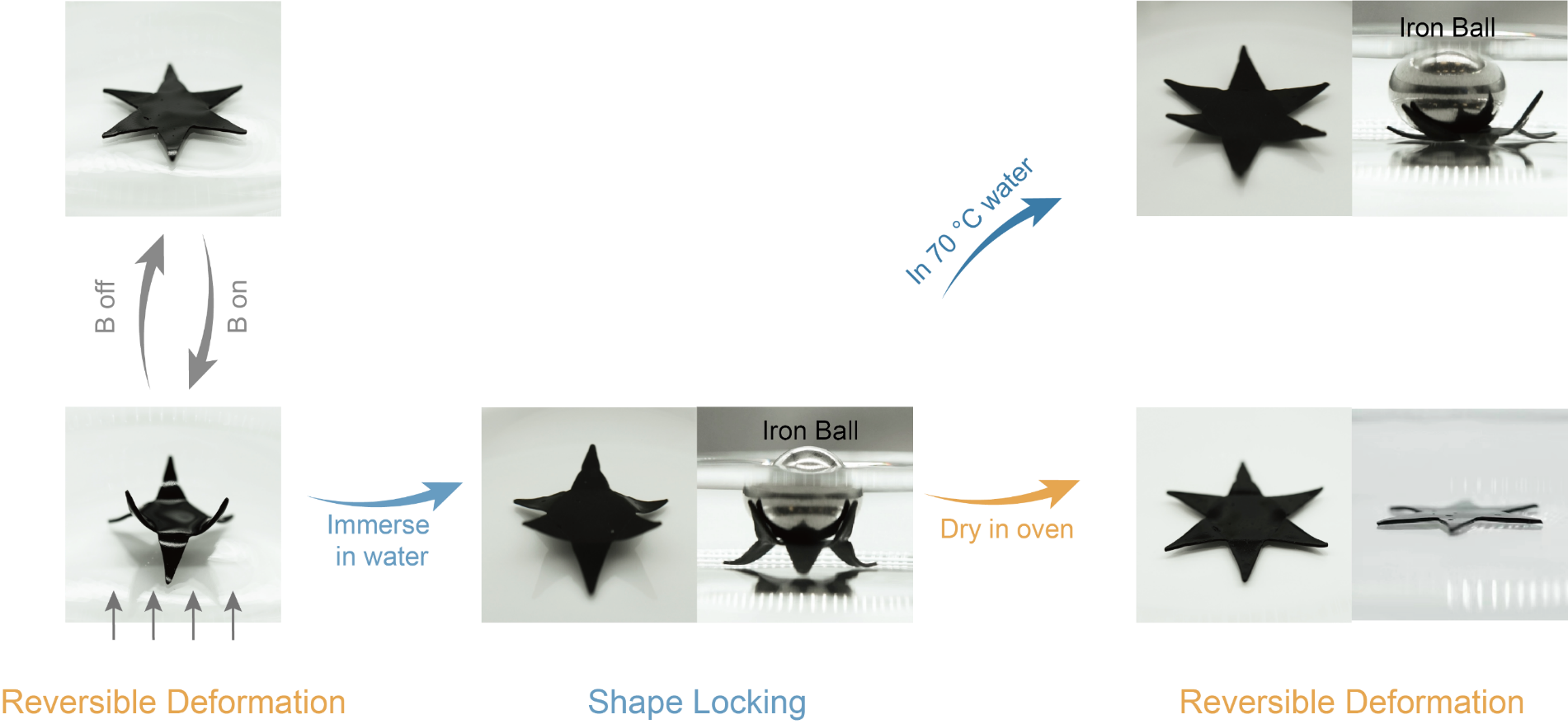


**Figure S10.** Digital photos showing the W-SML robot with a star-like pattern have reversible deformation under an external magnetic field and are locked into a chair-like structure after immersing in water. The locked W-SML robot can be unlocked by removing water through oven drying or by raising the temperature to 70 °C.


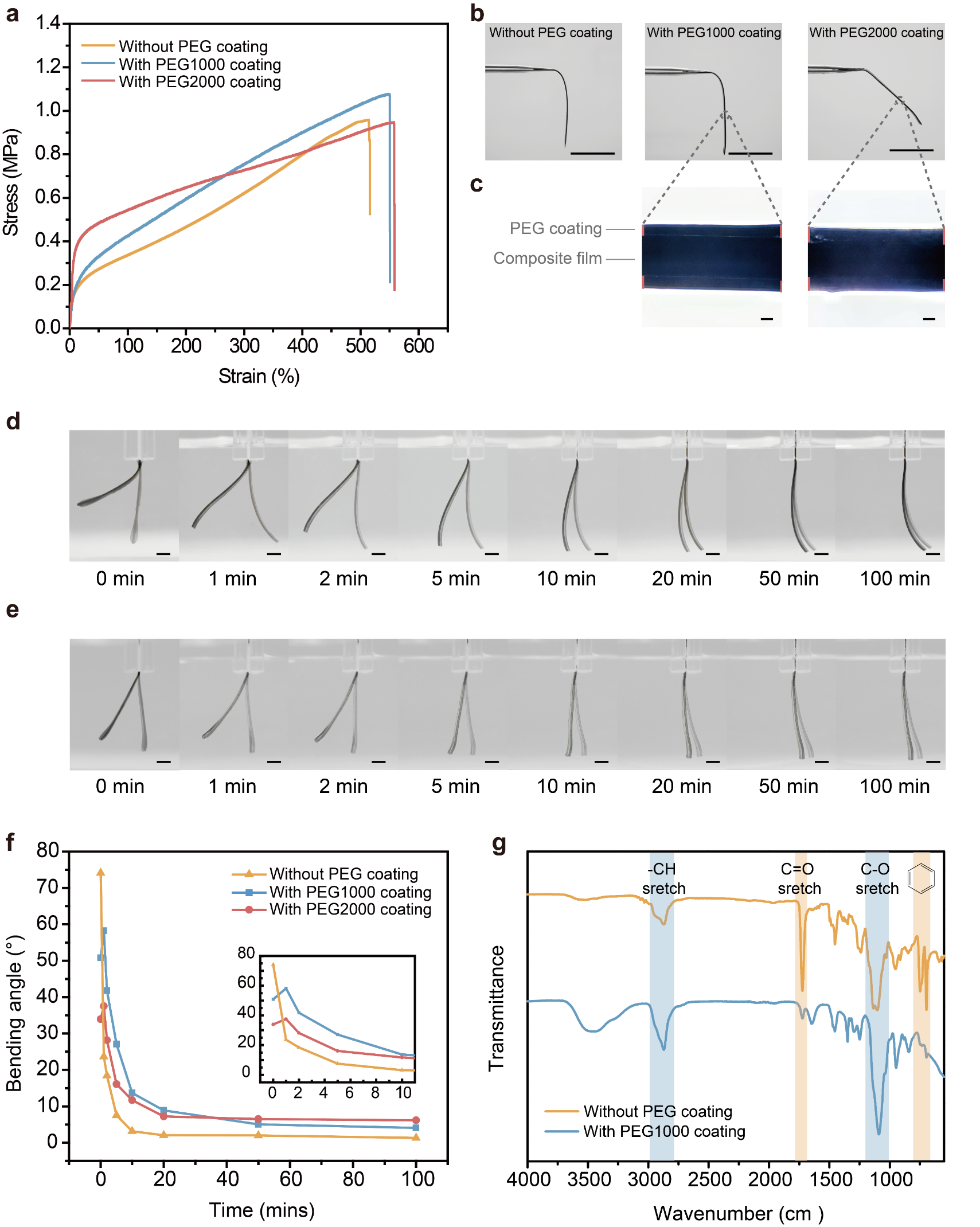


**Figure S11.** a) Tensile test results and b) optical images of the original NdFeB/P(BzMA-*co*-PEGMMA) composite film (weight ratio of 2:1) and composite films with PEG1000 and PEG2000 sacrifice layer at dry state. Scale bar: 2 cm. c) The cross-sectional optical microscope images showing the PEG1000 and PEG2000 sacrifice layers. Scale bar: 100 μm. d) Optical images showing the bending of the composite film with the PEG1000 sacrifice layer and e) with the PEG2000 sacrifice layer at different time scales after soaking. Scale bar: 2 mm. f) Bending angle of composite films as a function of water immersion time. g) FTIR spectra of composite films before and after coating with PEG1000 sacrifice layer.

The thickness of our composite film, approximately 350 µm, facilitates the rapid completion of the water absorption diffusion process, limiting the actuation performance of the sample quickly. To address this, we explored the use of PEG as a sacrifice layer. PEG was chosen for its excellent compatibility with the composite film (containing PEG side chains) and its properties as a biocompatible hydrophilic polymer that can act as a sacrificial layer to inhibit water diffusion into the composite film. This selection was aimed at prolonging the operation time without compromising the performance of the W-SLM robot. After coating the PEG1000 and PEG2000 layer, the modulus of the composite film increased from 1.78±0.21 MPa to 2.18±0.49 MPa and 3.87±0.52 MPa, respectively (Figure S11a, S11b). The corresponding thickness of PEG1000 and PEG2000 coating are around 110 and 130 µm, respectively (Figure S11c).

Figure S8c illustrates the actuation performance of composite films with PEG1000 and PEG2000 sacrificial layers. The dehydrated composite film with a PEG layer exhibited a lower bending angle than the sample without a PEG layer, which can be attributed to the inherent rigidity of PEG. During the initial 20 minutes of water immersion, the composite film with a PEG1000 layer demonstrated a higher bending ability than the one with a PEG2000 layer, attributed to the lower stiffness of PEG1000 itself compared to PEG2000. However, due to the higher chain length of PEG2000, it takes longer to completely dissolve. After nearly 40 minutes, the composite film with a PEG2000 layer exhibited a higher bending angle than the one with a PEG1000 layer. Given that our required operation time is within 20 minutes, we opted for PEG1000 as the sacrificial layer. FTIR analysis revealed an increased intensity of the characteristic peaks of PEG (C$-$H and C$-$H_2_ stretching, C$-$O stretching) and the almost disappeared characteristic peaks of BzMA (benzene ring bending), confirming the successful coating of PEG onto the composite film.


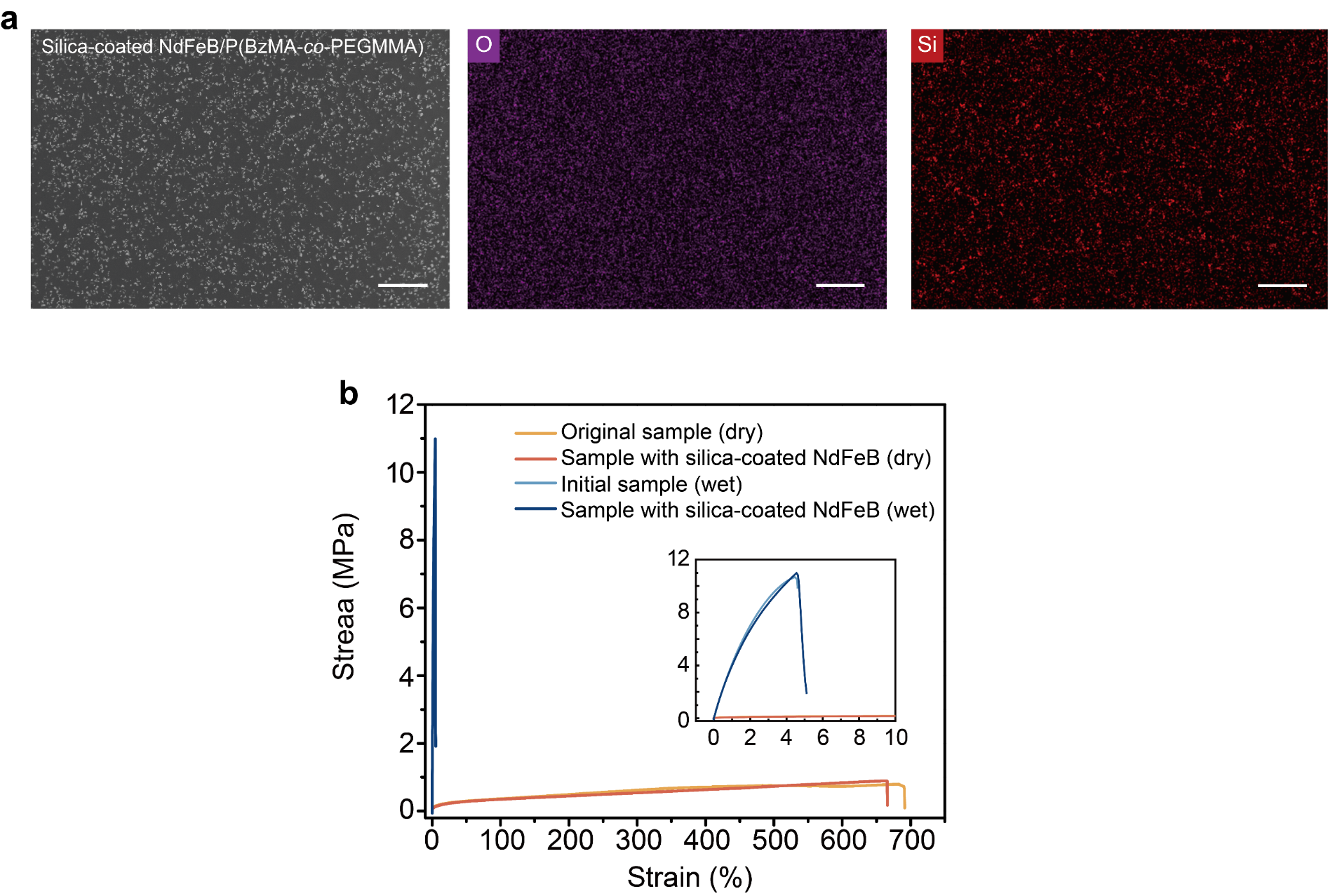


**Figure S12.** a) SEM image and corresponding element mapping of the surface of silica-coated NdFeB/P(BzMA-*co*-PEGMMA) composite film (weight ratio of 2:1). Scale bar: 100 μm. b) Tensile test results of the original NdFeB/P(BzMA-*co*-PEGMMA) composite film and silica-coated NdFeB/P(BzMA-*co*-PEGMMA) composite film.

The SEM image and corresponding element mapping of the surface of the silica-coated NdFeB/P(BzMA-*co*-PEGMMA) composite film confirm the successful coating of silica and reveal a similar particle distribution as in the composite film without silica coating. To investigate the influence of the silica coating on the mechanical properties of the polymer composite, tensile tests were conducted on both the composite film with silica-coated NdFeB and the original composite film. The results show almost identical tensile curves, indicating that the silica coating of NdFeB particles does not affect the mechanical properties of the polymer composite. This is likely because the silica coating does not alter the particle size or particle-matrix adhesion.


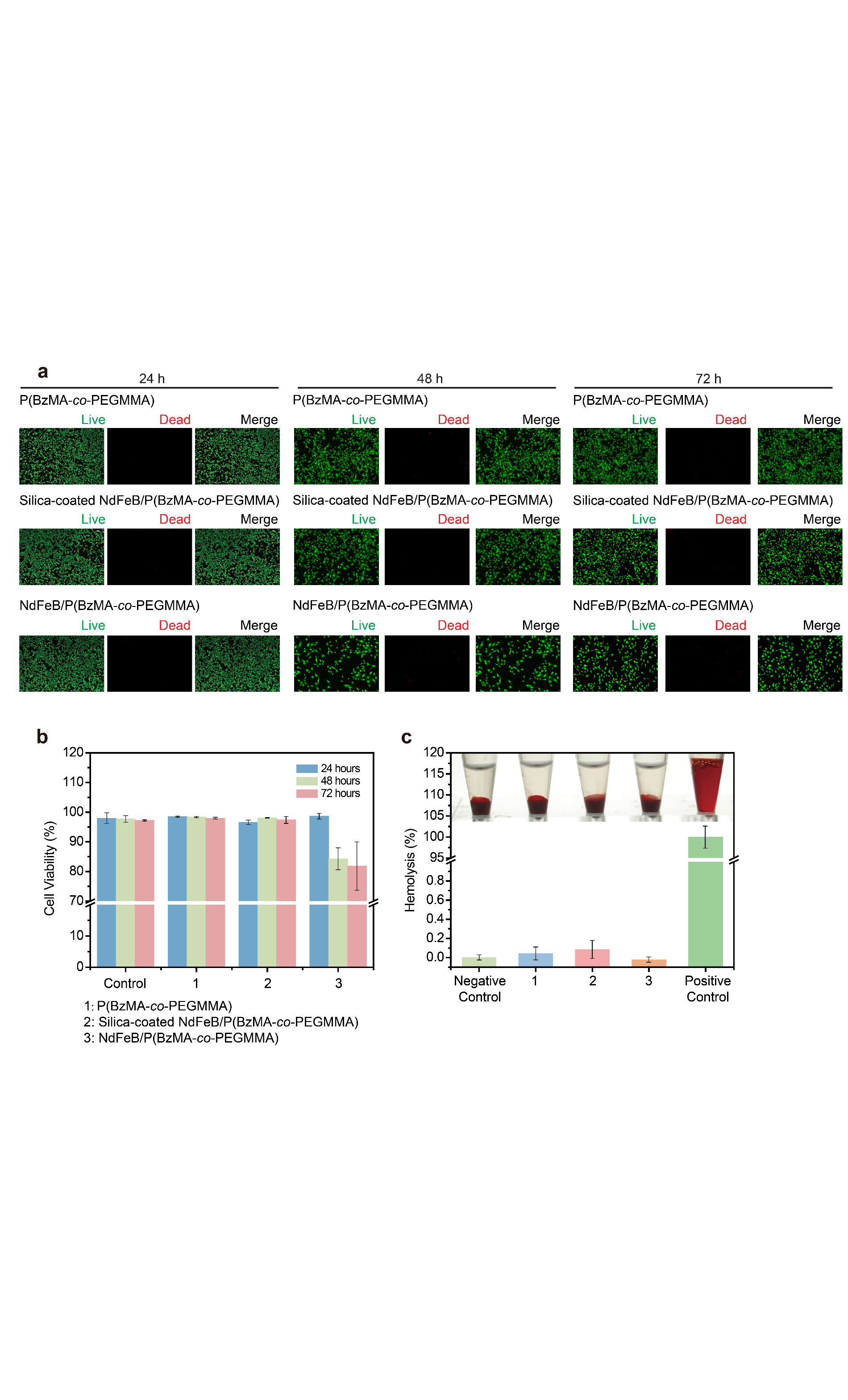


**Figure S13.** Cell viability tests and hemolytic tests of the water-stiffening polymer and polymer composites. a) Fluorescent image of HUVEC stained with Calcein/PI after 24-hour, 48-hour, and 72-hour co-culture with the polymer and polymer composites. b) Cell viability of HUVEC in four groups, i.e., control group, P(BzMA-*co*-PEGMMA), NdFeB/P(BzMA-*co*-PEGMMA), silica-coated NdFeB/P(BzMA-*co*-PEGMMA). c) Hemolysis of porcine blood cells in five groups, i.e., negative control, P(BzMA-*co*-PEGMMA), NdFeB/P(BzMA-co-PEGMMA), silica-coated NdFeB/P(BzMA-*co*-PEGMMA), positive control.

**Supplementary Tables and Table Captions**

**Table S1.** Comparison of potential body materials for magnetic robots with switchable modulus.

| Materials | Variation range | Stimulus | Shape locking | Wireless manipulation | Biocompatible |
| --- | --- | --- | --- | --- | --- |
| This work | 1.78 MPa-410 MPa | Water | Yes | Yes | Yes |
| SMP^[4]^ | 2.4 MPa-2.9 GPa | Heat  (20-100 °C) | Yes | Yes | N.A. |
| SMP^[5]^ | 3 MPa-3 GPa | Heat (50 °C) | Yes | No | Yes (Encapsulation) |
| LMPA^[6]^ | N.A.-3 GPa | Heat (47 °C) | Yes | No | Yes  (Encapsulation) |
| Electroactive gel^[7]^ | 20-240 kPa | Electric field | No | No | N.A. |
| Magnetorheological fluid^[8]^ | 187 Pa·s-1300 Pa·s | Magnetic field | No | Yes | N.A. |
| Magnetoactive liquid metal^[9]^ | 21.2 MPa-1.98 GPa | Heat  (30.6 °C) | Yes | Yes | N.A. |

**Table S2.** Young’s modulus, *T_g_* data of NdFeB/P(BzMA-*co*-PEGMMA) composite films at dry and wet state, and bending angle of dehydrated composite films with different NdFeB particles to P(BzMA-*co*-PEGMMA) weight ratio.

| NdFeB/P(BzMA-*co*-PEGMMA) ratio | $\sigma_{dry}$ (MPa) | $\sigma_{wet}$ (MPa) | Bending angle ($^{\circ}$) | *T_g_* at dry state ($℃$) | *T_g_* at wet state ($℃$) |
| --- | --- | --- | --- | --- | --- |
| 0 | 1.20±0.07 | 261±12 | 0 | -17.1 | 42.3 |
| 1:4 | 1.75±0.13 | 374±28 | 53.6±1.2 | -19.0 | 41.8 |
| 1:2 | 1.78±0.21 | 410±13 | 63.3±1.5 | -20.9 | 42.2 |
| 1:1 | 3.36±0.43 | 411±43 | 62.2±1.5 | -23.2 | 42.3 |
| 2:1 | 7.08±0.47 | 438±21 | 57.4±0.8 | -24.5 | 42.1 |
| 4:1 | 20.3±1.41 | 467±27 | 55.5±0.5 | -25.4 | 43.9 |

**Table S3.** Young’s modulus, elongation at break of the original NdFeB/P(BzMA-*co*-PEGMMA) composite film (weight ratio of 2:1) and healed composite film at dry state and after hydration (after immersing in water for 100 minutes) and the self-healing efficiency of the composite film.

|  | $\sigma_{original}$ (MPa) | $\varepsilon_{original}$ (%) | $\sigma_{healed}$ (MPa) | $\varepsilon_{healed}$ (%)^a^ | $\eta$ (%) |
| --- | --- | --- | --- | --- | --- |
| Dry state | 1.78±0.21 | 592±107 | 1.77±0.07 | 588±90 | 96.9 |
| Wet state | 410±13 | 4.40±2.63 | 414±29 | 2.94±1.16 | / |

^a)^ The self-healing efficiency of the composite film is calculated by the toughness of the healed sample over the initial Young’s modulus of the original sample.

**Supplementary Video Captions**

Video S1: Delivery and deployment of the robotic support (Figure 4b)

Video S2: Delivery and deployment of the robotic gripper (Figure 4d)

Video S3: Delivery and deployment of robotic helices (Figure 4f, 4g)

Video S4: Delivery and deployment of the stent (Figure S5e).**Reference**

[1] X. Liu, X. Dai, W. Boyko, A. S. Fleischer, G. Feng, *Colloids Surf. Physicochem. Eng. Asp.* **2022**, *633*, 127870.

[2] B. W. Chieng, I. N. Azowa, W. M. Z. W. Yunus, M. Z. Hussein, *Adv. Mater. Res.* **2014**, *1024*, 136.

[3] H. Yu, J. Wang, A. Natansohn, M. A. Singh, *Macromolecules* **1999**, *32*, 4365.

[4] Q. Ze, X. Kuang, S. Wu, J. Wong, S. M. Montgomery, R. Zhang, J. M. Kovitz, F. Yang, H. J. Qi, R. Zhao, *Adv. Mater.* **2020**, *32*, 1906657.

[5] M. Mattmann, C. De Marco, F. Briatico, S. Tagliabue, A. Colusso, X.-Z. Chen, J. Lussi, C. Chautems, S. Pané, B. Nelson, *Adv. Sci.* **2022**, *9*, 2103277.

[6] C. Chautems, A. Tonazzini, Q. Boehler, S. H. Jeong, D. Floreano, B. J. Nelson, *Adv. Intell. Syst.* **2020**, *2*, 1900086.

[7] Y. Li, Y. Maeda, M. Hashimoto, *Int. J. Adv. Robot. Syst.* **2015**, *12*, 175.

[8] Z. Chen, W. Lu, Y. Li, P. Liu, Y. Yang, L. Jiang, *ACS Appl. Mater. Interfaces* **2022**, *14*, 30007.

[9] Q. Wang, C. Pan, Y. Zhang, L. Peng, Z. Chen, C. Majidi, L. Jiang, *Matter* **2023**, *6*, 855.
